# The hyperthermophilic archaeon *Thermococcus kodakarensis* is resistant to pervasive negative supercoiling activity of DNA gyrase

**DOI:** 10.1093/nar/gkab869

**Published:** 2021-11-10

**Authors:** Paul Villain, Violette da Cunha, Etienne Villain, Patrick Forterre, Jacques Oberto, Ryan Catchpole, Tamara Basta

**Affiliations:** Université Paris-Saclay, CEA, CNRS, Institute for Integrative Biology of the Cell (I2BC), 91198 Gif-sur-Yvette, France; Université Paris-Saclay, CEA, CNRS, Institute for Integrative Biology of the Cell (I2BC), 91198 Gif-sur-Yvette, France; Immunology department, Institut Pasteur, Paris, France; Université Paris-Saclay, CEA, CNRS, Institute for Integrative Biology of the Cell (I2BC), 91198 Gif-sur-Yvette, France; Archaeal Virology Unit, Institut Pasteur, Paris, France; Université Paris-Saclay, CEA, CNRS, Institute for Integrative Biology of the Cell (I2BC), 91198 Gif-sur-Yvette, France; Université Paris-Saclay, CEA, CNRS, Institute for Integrative Biology of the Cell (I2BC), 91198 Gif-sur-Yvette, France; Department of Biochemistry and Molecular Biology, University of Georgia, Athens, GA 30602, USA; Université Paris-Saclay, CEA, CNRS, Institute for Integrative Biology of the Cell (I2BC), 91198 Gif-sur-Yvette, France

## Abstract

In all cells, DNA topoisomerases dynamically regulate DNA supercoiling allowing essential DNA processes such as transcription and replication to occur. How this complex system emerged in the course of evolution is poorly understood. Intriguingly, a single horizontal gene transfer event led to the successful establishment of bacterial gyrase in Archaea, but its emergent function remains a mystery. To better understand the challenges associated with the establishment of pervasive negative supercoiling activity, we expressed the gyrase of the bacterium *Thermotoga maritima* in a naïve archaeon *Thermococcus kodakarensis* which naturally has positively supercoiled DNA. We found that the gyrase was catalytically active in *T. kodakarensis* leading to strong negative supercoiling of plasmid DNA which was stably maintained over at least eighty generations. An increased sensitivity of gyrase-expressing *T. kodakarensis* to ciprofloxacin suggested that gyrase also modulated chromosomal topology. Accordingly, global transcriptome analyses revealed large scale gene expression deregulation and identified a subset of genes responding to the negative supercoiling activity of gyrase. Surprisingly, the artificially introduced dominant negative supercoiling activity did not have a measurable effect on *T. kodakarensis* growth rate. Our data suggest that gyrase can become established in *Thermococcales* archaea without critically interfering with DNA transaction processes.

## Graphical Abstract

**Graphical Abstract:**
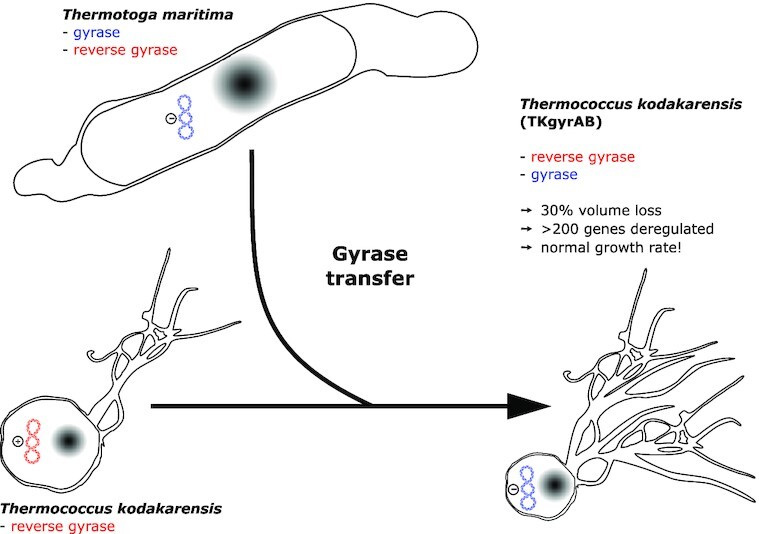
Introduction of DNA gyrase in Thermococcus kodakarensis flips its DNA supercoiling from positive to negative with only mild effects on its cell physiology.

## INTRODUCTION

With the discovery of the structure of DNA, it became apparent that the opening of the double helix generates torsional stress resulting in overwinding or underwinding of the DNA molecule (1). Paradoxically, many DNA transaction processes such as transcription and replication require strand separation and will lead naturally to DNA overwinding and strand entanglement (2–4). These topological constraints antagonise these essential cellular processes and if not resolved, are lethal. To deal with this problem, all cells rely on topoisomerases, a ubiquitous class of enzymes that introduce strand breaks to relieve unfavourable topological intermediates without damaging the genome (5–11). Topoisomerases are mechanistically classified as type I or type II, depending on whether they cleave one or two strands of DNA, respectively (6). Multiple phylogenetically unrelated subclasses of each type exist in the biosphere and such diversity has made it particularly challenging to dissect the evolutionary history of topoisomerases (12,13). A long-standing puzzle has been to understand why so many topoisomerases have emerged in the course of evolution and what role they played in the evolution of DNA-based cells (12,13).

DNA gyrase (hereafter gyrase), a type II A topoisomerase, is the only known enzyme that can negatively supercoil (underwind) DNA using the free energy of ATP hydrolysis to drive the process (14). An important antibiotic target, gyrase is essential and ubiquitous in bacteria where it controls (together with Topo I) the supercoiling density of chromosomes by introducing negative supercoils into DNA and by relaxing positive supercoils accumulating in front of moving DNA and RNA polymerases (15–18). The contribution of DNA gyrase in maintaining the chromosome in an underwound state in bacterial cells can profoundly impact the binding of regulatory proteins, promoter firing dynamics, DNA replication, and chromosome architecture (4,19,20).

DNA supercoiling is used in a wide range of bacteria to quickly transduce environmental signals towards the chromosome and this process is conserved in distant bacterial species (20,21). The most clearly described pathway involves the modulation of gyrase activity in response to [ATP]/[ADP] ratio in the cell. When this ratio is low, the DNA gyrase supercoiling activity is significantly reduced and the expression level of many genes is simultaneously modified (22,23). The inhibition of gyrase supercoiling activity by quinolone antibiotics has a similar effect; the expression of up to 48% of genes can be deregulated (21). This relatively simple, quick and general mechanism was suggested to be one of the key evolutionary inventions allowing the bacteria to occupy a wide variety of environments (12,13).

**Figure 1.**
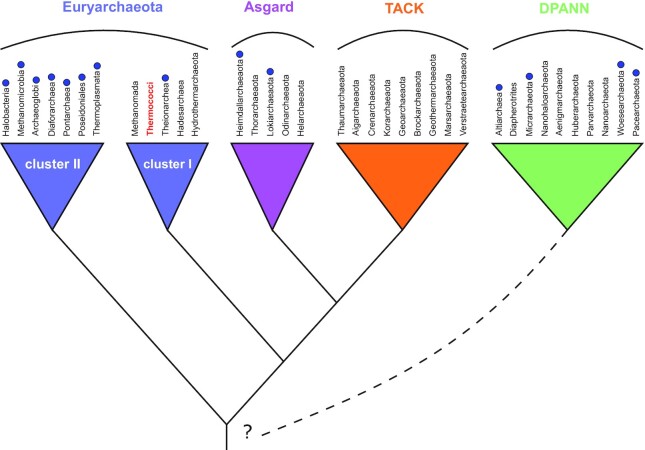
DNA gyrase distribution in Archaea. Schematic phylogeny of archaea with main phyla and superphyla indicated at the top. The presence of gyrase in a phylum is indicated by a blue dot. The model organism in this study, *Thermococcus kodakarenis* KOD1, belongs to Thermococci phylum within Euryarchaeota I clade. The dashed line symbolizes the uncertainty of DPANN branching within the Archaea tree.

In Archaea, Gyrase is found in all members of the highly diversified monophyletic group (named Cluster II Euryarchaeota by Adam and colleagues) containing seven distinct groups with very different lifestyles (acidophiles, halophiles, methanogens among others) and, sporadically, in DPANN and Asgard superphyla (12,24,25) (Figure 1). Initial phylogenetic analyses indicated that archaeal gyrase is of bacterial origin and was acquired via ancient horizontal gene transfer by a hyperthermophilic archaeon (12). Later analysis including more archaeal lineages suggested that this transfer occurred only once at the base of the aforementioned late emerging Cluster II Euryarchaeota (26). Because negative supercoiling facilitates DNA melting, it was proposed that gyrase acquisition had a profound impact on all DNA-dependent processes with important consequences for the evolution of recipient archaea (13,27,28). However, how and why DNA gyrase became fixed in archaeal lineages remains obscure.

The successful establishment of bacterial gyrase in Archaea is particularly intriguing since the introduction of an uncontrolled negative supercoiling activity could potentially interfere with all DNA-templated processes. To make things worse, the recipient archaeon was probably a thermophile (28–31) and therefore encoded a reverse gyrase with opposite, positive supercoiling activity which is essential for life at high temperature (32–35). Finally, archaea encode Topo VI as the main type II topoisomerase and its predicted *in vivo* role in relaxing the positive supercoils overlaps with that of gyrase (36).

To understand better the challenges imposed by DNA gyrase to a naïve archaeal cell we introduced a bacterial gyrase in the genetically tractable hyperthermophilic archaeon *Thermococcus kodakarensis* TS559 which naturally has slightly positively supercoiled DNA (37). This archaeon encodes histones and three topoisomerases (reverse gyrase, Topo III and Topo VI) thus mimicking the topological ‘kit’ present in the ancestor of the Cluster II Euryarchaeota (13,29). As a source of gyrase, we selected the one from the bacterium *Thermotoga maritima* (TmGyrAB) since its closest relatives are archaeal gyrases (Villain *et al.*, unpublished) increasing the chance that its activity would not be impaired by the archaeal cellular context. In addition, the optimal growth temperature of *T. maritima* (80°C) and that of *T. kodakarensis* (85°C) are similar, both were isolated from geothermally heated sea floors (38,39) and they can be co-cultured in laboratory. Finally, TmGyrAB exhibits the expected negative supercoiling activity both *in vitro* and *in vivo* (40–42).

We found that the gyrase of *T. maritima* was active and predominant in *T. kodakarensis* such that the normally positively supercoiled plasmid DNA was converted to strongly negatively supercoiled DNA. Gyrase interacted with the genome of *T. kodakarensis* and induced differential expression of hundreds of genes, a subset of which specifically responded to negative supercoiling activity. Reverse gyrase was the only topoisomerase that reacted transcriptionally, albeit modestly, to negative supercoiling activity. Despite unnatural gyrase-enforced topological changes, the growth of *T. kodakarensis* was not affected. We conclude that gyrase is remarkably well tolerated by *T. kodakarensis* suggesting the existence of resilience mechanisms against torsional stress which may have been instrumental for the natural establishment of gyrase in the archaeal domain.

## MATERIALS AND METHODS

### Construction of recombinant *Thermococcus kodakarensis* TS559 strains

All the strains, plasmids and oligonucleotides used in this study are listed in the Supplementary table S1 and S2. Plasmids were constructed in *Escherichia coli* strain XL1-Blue grown at 37°C in LB supplemented with Ampicillin (100 μg/ml), Kanamycin (40 μg/ml) or Chloramphenicol (20 μg/ml) using standard molecular biology protocols. The gyrase-encoding genes *gyrA* and *gyrB* were PCR amplified using genomic DNA of *Thermotoga maritima* MSB8 as template and cloned as a bi-cistronic operon in plasmid pTNAg (43) under the control of the strong constitutive promoter PhmtB. Plasmid constructions were performed by Gibson Assembly using the NEBuilder HiFi DNA Assembly Master Mix (New England Biolabs) following the manufacturer's protocol.

*T. kodakarensis* TS559 was transformed using standard protocol in anaerobic conditions using the rich medium ASW-YT or the synthetic medium without tryptophan ASW-AAW (43,44). Briefly, 10 ml of late exponential phase culture was pelleted and resuspended in 100 μl of 0.8× ASW. At least 2 μg of plasmid DNA was mixed with 100 μl of cell suspension and incubated on ice for 1 h, heat shocked for 1 min at 85°C and cooled on ice for 10 min. Then 1 ml of non-selective medium was added and cell suspensions were incubated 90 min at 85°C for recovery. Cells were then harvested by centrifuging 4 min at 4500 g, resuspended in 200 μl of 0.8× ASW and plated onto selective solid medium containing 1% (w/v) Phytagel™ (Sigma-Aldrich). Plates were reduced with 2 ml of polysulfide solution (100 g of Na_2_S nonahydrate and 30 g of sulfur dissolved in 75 ml of water) per litre of medium and starch azure was added at 0.2% (w/v) to facilitate visualisation of colonies (45). After 40 h (ASW-YT) or 64 h (ASW-AAW^–^) of incubation at 85°C isolated colonies were transferred to selective liquid medium in sealed bottles under N_2_ atmosphere. Na_2_S was added to a final concentration of 0.02% (w/v) and resazurin was added at 1 mg/l as an indicator of medium reduction.

To measure growth, liquid cultures of *T. kodakarensis* were inoculated at 1:100 dilution from fresh precultures. Cell density was monitored with phase-contrast microscopy using a Thoma cell counting chamber (0.01 mm depth) or using an optical device that measured turbidity variations directly in Hungate tubes (MicrobeMeter).

The expression of the *gyrA* and *gyrB* genes in transformants was tested by RT-PCR. Briefly, three individual clones of *T. kodakarensis* TS559 were each grown in 25 ml of ASW-YT without agmatine until late exponential phase. Cells were harvested (5000 × g, 10 min) and resuspended in 500 μl of resuspension buffer (NaCl 1 M, Tris HCl 0.1 M, CaCl_2_ 5 mM, MgSO_4_ 0.1 M). The RNAs were extracted using TRIzol according to the supplied protocol (Sigma-Aldrich). To eliminate residual DNA, the samples were treated for 30 min at 37°C with TURBO DNase (ThermoFisher) and reextracted with TRIzol. The cDNA was synthetized using Maxima First Strand cDNA Synthesis kit (ThermoFisher) following the manufacturer's protocol. The PCR reactions were performed using each cDNA or total RNA sample as template. The used oligonucleotides are listed in Supplementary table S2. The obtained PCR products were separated on 1% (w/v) agarose gel and stained with ethidium bromide (0.5 μg/ml).

### Wide-field microscopy and DNA staining of *T. kodakarensis* cells

A small volume (200 μl) of exponentially growing culture was rapidly cooled down in mixture of ice and water to limit the effects of cold shock and oxygen exposure during imaging. Cells were then centrifuged and resuspended in an equal volume of 0.8× ASW containing 1 μg/ml of Hoechst 33342 (excitation 361 nm / emission 486 nm) for DNA staining. Cells were incubated for 10 min in the dark and then mounted onto glass slides covered with a thin layer of 1% agarose (suspended in 0.8× ASW solution). DIC and fluorescent images were obtained at room temperature on an SP8 confocal laser scanning microscope (Leica Microsystems) equipped with hybrid detectors and a 63x oil immersion objective (HC Plan Apo, 1.4 numerical aperture [NA]; Leica). Fluorescence detection was performed by exciting the sample with a 405 nm laser and collecting fluorescence between 415 and 515 nm) at the speed of 600 Hz with a line averaging of three. Image format was adjusted to provide an XY optimal sampling (pixel size of 60 nm) and for each position z-stacks (3 μm width and 0.5 μm step) were acquired.

Method Yen from MicrobeJ software (46) was used to perform automatic cell detection and size measurements. The obtained profiles were manually curated to remove debris and aggregated cells. For cell size, circularity and area measurements at least 50 independent images were analysed per strains totalling at least 600 analysed cells.

### Two-dimensional agarose gel electrophoresis

Plasmids pTNAg-Y119F and pTNAg-gyrAB are too large (12 602 bp) to be easily resolved by 2D agarose gel electrophoresis we instead used the smaller reporter plasmid pTPTK2 (5455 bp) (43). *T. kodakarensis* strains were grown in Ravot medium (47) to increase the yield of intact supercoiled plasmids.

Ravot medium was inoculated from overnight precultures grown in ASW-AAW medium at 1:100 dilution, and growth was monitored until late exponential phase. Then cultures were rapidly chilled in precooled beaker immersed in a water-ice bath to stop topoisomerase or nuclease activities. Plasmids were extracted with the NucleoSpin^®^ kit (Macherey-Nagel) following the low copy manufacturer's protocol with minor modifications: (i) the lysis step was reduced to 1 min to limit plasmid nicking, (ii) two steps of lysate clarification were performed and (iii) the wash step (AW) was performed to inactivate residual nucleases.

Agarose gels were prepared by dissolving 0.8% (w/v) of ultrapure agarose (Invitrogen) in 1× TBE buffer (89 mM Tris, 89 mM boric acid, 2 mM EDTA) and pouring in 14.5 × 20 cm tray. Electrophoresis was performed at 24°C and minimum 3 μg of plasmid DNA was used for each sample. In the first dimension, no intercalating agent or 1.5 μg/ml of chloroquine (Alpha Aesar) was added in the gel and in the running buffer. Electrophoresis was run at 1.2 V/cm for 40 h. Gels were subsequently equilibrated for 1 h in 1× TBE buffer supplemented with 7.5 μg/ml of chloroquine and placed in the tray after a rotation of 90°. In the second dimension, an electric field of 2 V/cm was applied for 10 h. Gels were washed 3 × 30 min in water to remove chloroquine and then stained for 1 h with 2.5 μg/ml of ethidium bromide. Gels were then rinsed with water and imaged with a Typhoon Imager (Amersham) using Cy3 channel.

### Calculation of the supercoiling density

**Figure 2.**
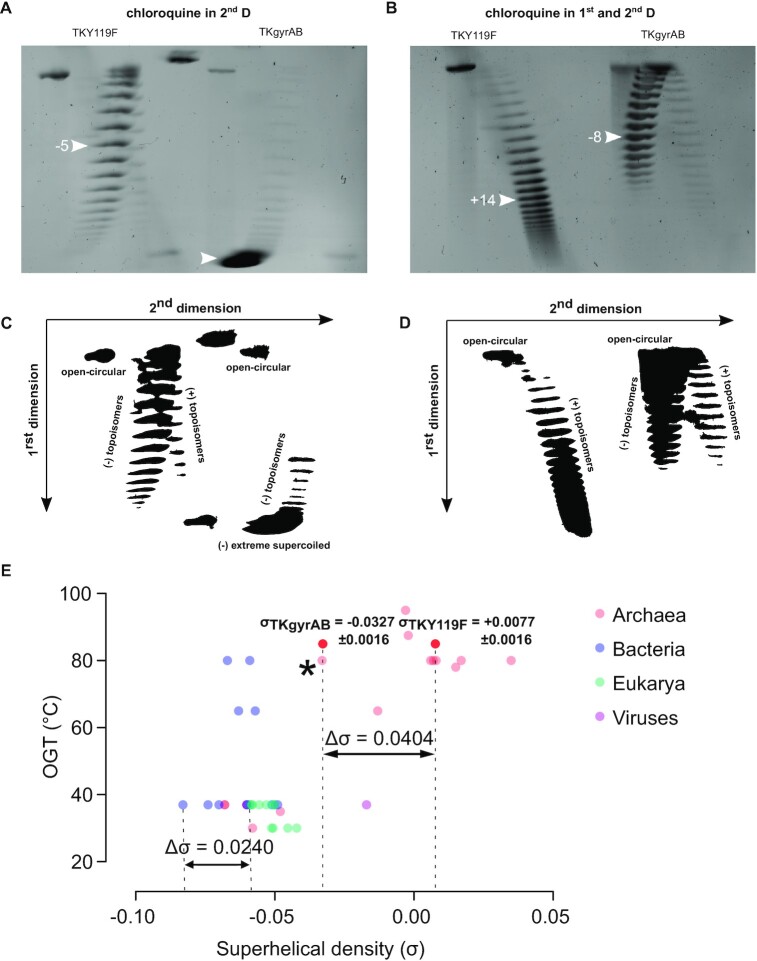
Plasmid DNA from *Thermococcus kodakarensis* TKgyrAB is negatively supercoiled. Reporter plasmid pTPTK2 was isolated from TKY119F or TKgyrAB strains and its topoisomers were separated using 2D agarose gel electrophoresis. The corresponding cartoon is depicted below each agarose gel. (**A**) and (**C**) DNA intercalating drug chloroquine was added at 7.5 μg/ml only in the second-dimension run. This allows to separate positively and negatively supercoiled topoisomers as fast-migrating right arc and slow-migrating left arc, respectively. Under these running conditions, the majority of pTPTK2 isolated from TKgyrAB strain is in extremely negatively supercoiled form. The major (the most abundant) topoisomer from the TKY119F is indicated with a white arrow and was used to calculate superhelical density. (**B** and **D**) Chloroquine was added both in the first (1.5 μg/ml) and the second-dimension run (7.5 μg/ml). Chloroquine introduces a slight positive torsion in DNA, thus changing the apparent superhelical density of topoisomers toward a positively supercoiled state. This allowed relaxing the extremely negatively supercoiled form of pTPTK2 during the first dimension run such that the individual topoisomers could be separated and the major (the most abundant) topoisomer determined. This topoisomer is indicated by a white arrow and was used to calculate superhelical density. (**E**) Plasmid superhelical densities from various organisms were plotted against the optimal growth temperature of their hosts (see also supplementary table S3). The mean σ and standard deviation calculated from three independent experiments are indicated for gyrase expressing strain TKgyrAB and the control strains TKY119F. The point corresponding to Archaeoglobi archaea is highlighted by an asterisk. The total change in the supercoiling density (Δσ) resulting from gyrase activity in *T. kodakarensis* or from TopoI inhibition in *Streptococcus pneumonia* is indicated.

Superhelical density (σ) of the reporter plasmid pTPTK2 (5455 bp) was calculated from the imaged 2D gels using an adaptation of the band-counting method as described by López-García and Forterre (48). As an example, we detail the calculation for the gels presented in Figure 2. Topoisomers were first separated without chloroquine in the first dimension and the major topoisomer was identified using band intensity measurement with Fiji software (49). This gave the writhe for the plasmid pTPTK2 (Wr = –5) in strain TKY119F. A second gel was run with 1.5 μg/ml of chloroquine in the first dimension, allowing to determine a Wr = + 14 for plasmid pTPTK2 in strain TKY119F. We thus determined that the chloroquine introduced 19 positive supercoils in pTPTK2. To account for the temperature effect on plasmid topology between the growth temperature of *Thermococcus* (85°C) and the electrophoresis (24°C) we applied the correcting factor of - 0.011°/°C/bp (50). The temperature difference thus introduced 10 negative supercoils in pTPTK2. Taking into account the chloroquine effect in the first dimension and the unwinding induced by temperature, we determined a Wr of –5 for the TKY119F strain (Wr = 14 + 10 – 19) and -17 for the TKgyrAB strain (–8 + 10 – 19). Tw is equal to the length of the plasmid molecule (in base pairs) divided by the number of base pairs per helix turn (h) (h = 10.5 bp per turn under standard conditions). For pTPTK2 the twist is Tw = 5455 bp/10.5 bp. Finally, the σ was calculated by dividing the Wr by Tw to yield σ_TKY119F_ = +0.0096 and σ_TKgyrAB_ = –0.0327. The mean σ values were calculated from three independent 2D gel electrophoresis experiments.

### Plasmid toxicity assay

*T. kodakarensis* strains TKY119F and TKgyrAB, carrying respectively pLCAg-Y119F and pLCAg-gyrAB, were passaged 14 times at 85°C with 100-fold dilution in Ravot medium or in Ravot medium supplemented with 1 mM of agmatine. The culture used for the first inoculation and the 4th, 9th and 14th subcultures, corresponding respectively to 0, 24, 54 and 84 cell generations, were anaerobically sampled. These samples were 10-fold serial diluted in 1X Ravot salts solution to a 10^–7^ dilution. 10 μl of each dilution were spotted on solid selective (Ravot) or non-selective (Ravot with agmatine) medium. Plates were reduced with polysulfides solution and supplemented with starch azure as described above. After 2 days of incubation in anaerobic jars at 85°C, cell viability was determined by counting colonies from spotted dilutions.

### Ciprofloxacin susceptibility test

Ciprofloxacin sensitivity of TKY119F and TKgyrAB was investigated on plates using an adaptation of the inverted spot test method (47). Briefly, in anaerobic conditions, 25 ml of Ravot-phytagel medium supplemented with agmatine was poured in Petri dishes as described above. On this bottom layer, a 5 ml thin layer composed of 1X Ravot medium, 0.18% (w/v) of Phytagel™, 1 mM of agmatine, 1% of colloidal sulfur and 160 μl of late exponential phase *T. kodakarensis* culture was poured. On the solidified top layer, 5 mm Whatman paper discs were gently laid. 10 μl of ciprofloxacin dilutions dissolved in 0.1 N HCl were spotted on the paper discs. On such plates the top layer initially appears milky-white because of the colloidal sulfur. Growing *T. kodakarensis* consumes the sulfur and the top layer becomes transparent. After overnight incubation in anaerobic jars, growth inhibition was assessed by measuring the diameter of the remaining milky-white halos around the Whatman paper discs.

### Differential gene expression analysis

To prepare samples for RNA sequencing, 25 ml of Ravot medium was inoculated at 1/100 dilution with fresh *T. kodakarensis* preculture. Total RNA was extracted from 20 ml of exponentially growing cultures (6 h of culture, approximately 2 × 10^9^ cells/ml) using a NucleoSpin RNA set for NucleoZOL (Macherey Nagel). DNA was eliminated from samples using a TURBO DNA-free kit following the manufacturers protocol (Ambion). For each strain, biological replicates were prepared from four independent cultures. Total RNA quality was assessed on an Agilent Bioanalyzer 2100, using RNA 6000 pico kit (Agilent Technologies). Directional RNA-Seq Libraries were constructed using the TruSeq Stranded Total RNA library prep kit, with bacteria Ribo-Zero reagents (Illumina), following the manufacturer's instructions; 500 ng of total RNA were used. After the Ribo-Zero step, the samples were checked on the Agilent Bioanalyzer for proper rRNA depletion. Final libraries quality was assessed on an Agilent Bioanalyzer 2100, using an Agilent High Sensitivity DNA Kit. Libraries were pooled in equimolar proportions and sequenced on a Paired-End 2 × 75 bp run, on an Illumina NextSeq500 instrument. Demultiplexing was performed with bcl2fastq2 v2.18.12. Adapters were trimmed with Cutadapt v1.15, and only reads longer than 10 bp were kept for further analysis. Between 32 and 56 million reads for each sample were mapped on genome of *T. kodakarensis* TS559 and plasmid sequences with Burrow-Wheeler Aligner for short-read alignment release 0.7.17-r1188 (51). Reads per gene were counted using subread featureCounts v1.5.2 and differential analyses were performed in R using DESeq2 v1.28.1 package (52).

Adjusted *P*-values histograms, Principal Component Analysis, Volcano Plot, Heatmap and distribution of deregulated genes across *T. kodakarensis* TS559 chromosome were drawn using R with gplots and ggplot2 packages. COGs categories of *T. kodakarensis* genes were extracted from ‘The ArCOG database’ (53) and used to draw the deviation graph with the plotrix R package.

## RESULTS

### Construction of gyrase-expressing *Thermococcus kodakarensis* strains

We transformed *T. kodakarensis* with the replicative plasmid pTNAg encoding *gyrA* and *gyrB* genes from *T. maritima*. The genes were expressed from *gyrAB* operon under the control of the constitutive archaeal promoter PhmtB. Single clone transformants that we named *T. kodakarensis* TKgyrAB strain (hereafter TKgyrAB) were readily obtained and the expression of the *gyrAB* was confirmed by RT-PCR (Supplementary figure S1). We also constructed a control strain carrying the empty vector (hereafter TKAg) and the strain expressing a mutated version of the gyrase where the catalytic tyrosine 119 was replaced by phenylalanine (hereafter TKY119F). This gyrase mutant was shown to bind DNA and ATP but is unable to generate double strand breaks necessary to introduce negative supercoiling in the DNA (54,55). We could not detect protein bands (90.5 kDa for GyrA and 72.5 for GyrB) corresponding to gyrase subunits on a SDS-PAGE gel indicating that in this experimental setup the gyrase was not expressed to very high levels compared to native proteins in TKgyrAB and TKY119F strains (Supplementary figure S2).

### Plasmid DNA is negatively supercoiled in gyrase-expressing *T. kodakarensis*

Native plasmids of *Thermococcus* species are positively supercoiled *in vivo* presumably by the action of reverse gyrase (48). To investigate the impact of gyrase on DNA topology in *T. kodakarensis* we analysed the topology of plasmids isolated from the TKGyrAB, TKAg or TKY119F cultures using agarose gel electrophoresis. To facilitate these analyses, we introduced a smaller reporter plasmid pTPTK2 (5455 bp) into each of the three recombinant *Thermococcus* strains.

Plasmid topoisomers can be separated as single bands by one dimensional slow electrophoresis in agarose gels containing no DNA intercalating agent. In such conditions, the most highly supercoiled plasmids migrate the fastest forming a single front-band. We observed a broad range distribution of topoisomers in the two control strains as expected for plasmids with low level of positive supercoiling and only one fast-migrating major band of highly supercoiled DNA in the TKGyrAB strain (Supplementary figure S3A). This suggested that the gyrase was active in *T. kodakarensis*.

To confirm that the fast-migrating major band corresponded to negatively supercoiled DNA (in 1D electrophoresis, positively and negatively supercoiled topoisomers behave identically), we performed 2D agarose gel electrophoresis to determine the orientation (either positive or negative) of the supercoiling (56).

Initial 2D gel electrophoresis was performed in the absence of chloroquine in the first dimension to preserve the natural plasmid topology and with 7.5 μg/ml of chloroquine in the second dimension to determine the direction of supercoiling. This confirmed that the vast majority of the reporter plasmid was in an extreme negatively supercoiled state, in stark contrast to plasmid isolated from the control TKY119F strain (Figure 2A and C). The distribution of the topoisomers served as a reference to determine the number of supercoils introduced by chloroquine. Next, the plasmids were separated by adding chloroquine in both the first and the second dimension. This allowed us to determine the ΔLk for the major topoisomer in the gyrase expressing TKgyrAB strain (ΔLk = –8) as well as in the control strain TKY119F (ΔLk = –5) by taking into account the number of positive supercoils (+19) introduced by intercalation of chloroquine (Figure 2B and D). We used these values to calculate the mean supercoiling density from three independent experiments, after correction for the temperature effect (due to helix pitch increase) on the plasmid topology (–10 supercoils) we obtained a superhelical density of +0.0077 ± 0.0016 for the control strain (σ_TKY119F_) and –0.0327 ± 0.0016 for the strain containing the active DNA gyrase (σ_TKgyrAB_).

Supercoiling density is used as a standardized measure of linking difference and can be compared between different organisms regardless of plasmid size or culturing conditions. The determined mean σ_TKY119F_ was identical to that of the strain TKAg (Supplementary figure S3B) and it matched well with the reported native plasmid supercoiling level of *Thermococcus* sp. (48,57), thus suggesting that the inactive gyrase did not introduce topological changes in the reporter plasmid DNA (Figure 2E, supplementary table S3). The supercoiling density of gyrase-expressing *T. kodakarensis* matched that of Archaeoglobi archaea which are the only gyrase-encoding hyperthermophilic archaea (Figure 2E, asterisk, Supplementary table S3) (58). Remarkably, the increase in negative supercoiling density in TKgyrAB strain as compared to the control strain is more than 5-fold.

Collectively, the data show that active gyrase can be expressed in *Thermococcus kodakarensis* and that this organism can tolerate a substantial increase in negative supercoiling of its plasmid DNA. The data also suggest that the endogenous topoisomerases of *T. kodakarensis* with capacity to relax negatively supercoiled DNA (principally reverse gyrase and to a lesser extent Topo III and Topo VI) were outcompeted by gyrase.

### The gyrase-expressing *T. kodakarensis* is sensitive to ciprofloxacin

We next asked if gyrase interacted with chromosomal DNA of *T. kodakarensis* and catalysed the double-stranded breaks required to introduce negative supercoiling. This can be tested indirectly by measuring the sensitivity of strains to the antibiotic ciprofloxacin. Ciprofloxacin binds to gyrase and DNA (59) and kills bacteria efficiently by stabilising the double-stranded break occurring during gyrase activity (60,61).

**Figure 3.**
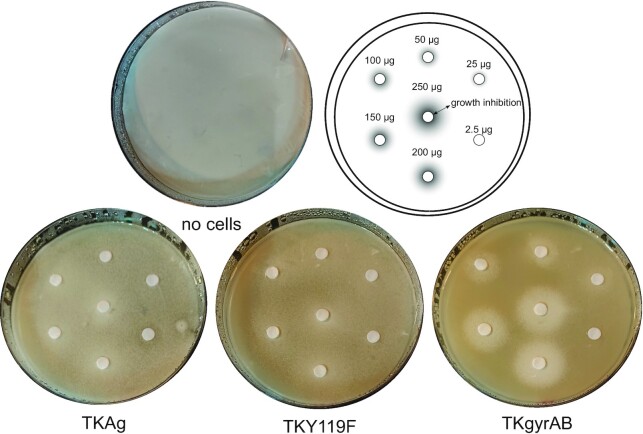
Gyrase expression induces ciprofloxacin sensitivity in *T. kodakarensis Thermococcus* cells from overnight cultures were mixed with 0.18% (w/v) phytagel and colloidal sulfur containing culture medium and were spread as overlay onto 1% phytagel plates. The plate cartoon indicates the position of the paper discs and the quantity of ciprofloxacin used. The cell-containing overlay becomes transparent when incubated at 85°C due to consumption of colloidal sulphur during *T. kodakarensis* growth. Growth inhibition can therefore be detected as a white area on the plate.

In contrast to what is reported for bacteria, we had to use relatively high concentrations of the drug to observe growth inhibition on plates (Figure 3). Since less than 10% of ciprofloxacin is degraded at the incubation temperature and time-scales we used (62), the low sensitivity of strain TKGyrAB to the drug may be explained by inefficient transfer of the drug across the membrane and/or very efficient efflux pumps. Intriguingly, we also observed a significant growth retardation of our control strains when exposed to ciprofloxacin indicating that the drug interferes with essential process(es) in *T. kodakarensis*. The figure 3 shows the result of the antibiogram test on phytagel plates. The cells were plated on non-selective medium to make sure that we detected the toxic effect of the ciprofloxacin (gyrase-DNA covalent adducts) against the chromosome and not against the plasmid that carried the selective marker. The assay repeatedly (*n* = 4) showed higher sensitivity of TKGyrAB strain to the ciprofloxacin compared to controls, consistent with the formation of toxic gyrase-DNA covalent adducts on the chromosome.

### Global transcriptional response to gyrase expression in *Thermococcus kodakarensis*

To understand better how *T. kodakarensis* cells cope with the presence of artificial negative supercoiling activity we investigated genome-wide transcriptional responses in the three recombinant strains.

We performed RNA-seq experiments on biological replicates (*n* = 4) of exponentially growing cells and quantified differential transcript abundance in TKgyrAB versus TKAg, TKY119F versus TKAg and TKgyrAB versus TKY119F (Supplementary figure S4). We reasoned that the latter analysis would, in principle, allow us to identify the subset of genes enriched for those responding specifically to the negative supercoiling activity of the gyrase while TKY119F versus TKAg comparison would yield genes responding to the burden of gyrase heterologous expression and its DNA binding activity. In agreement with this hypothesis, only few differentially expressed genes (DEGs) are shared between TKgyrAB vs TKY119F (genes responding to negative supercoiling) and TKY119F versus TKAg (genes responding to gyrase expression burden and DNA binding) or in all three comparisons (Supplementary figure S5).

We first compared the transcriptional profile of the genes encoded on the empty vector and the two plasmids carrying gyrase genes. This was particularly interesting because, in contrast to the chromosome where the local DNA topology status was not known, we knew that the plasmid encoding active gyrase was negatively supercoiled and thus we could measure how negative supercoiling affected gene transcription. Negative supercoiling facilitates DNA melting and thereby promotes transcription (63,64). We therefore anticipated an increase in expression of plasmid-borne genes when comparing TKgyrAB vs TKY119F transcriptional profiles. Intriguingly, however, none of the genes (including the *gyrAB* operon) were differentially expressed (Supplementary table S4). Interestingly, the expression of inactive gyrase was not neutral, three out of five plasmid-encoded genes were significantly downregulated suggesting that gyrase might bind within or in the vicinity of these genes and interfere with their transcription. It has to be noted though that the interpretation of these data needs to be taken with precaution since the precise plasmid copy number in each strain is not known.

**Figure 4.**
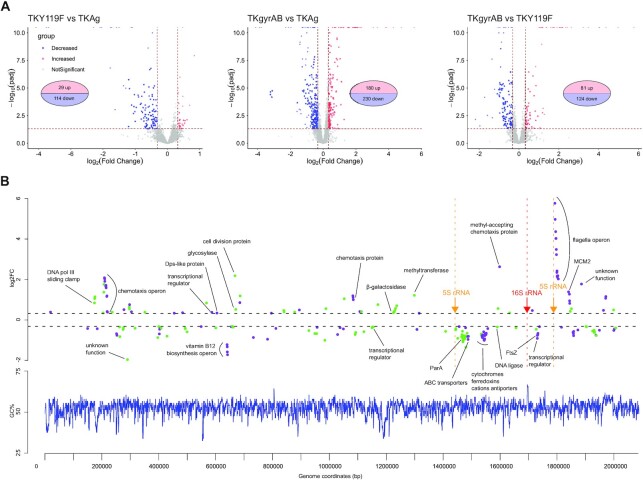
The impact of gyrase on transcription in *T. kodakarensis* (**A**) Volcano plots showing significantly deregulated genes. The two vertical and the horizontal dashed lines indicate the threshold values of ±1.25 fold change and Padj > 0.05, respectively. The blue and red dots correspond to downregulated and upregulated genes, respectively. Each plot corresponds to a pairwise comparison of transcriptomes from strains indicated on the top. (**B**) Distribution of the SRGs on the chromosome of *T. kodakarensis* TS599. Top panel shows the distribution of DEGs on the chromosome of *T. kodakarensis*. Points correspond to individual DEGs and their colour indicates the orientation of transcription whereby green indicates antisense expressed genes and violet indicates sense expressed genes. The position of ribosomal genes is indicated by arrows. The annotated function of SRGs involved in DNA transactions and cell division (COG categories L, K, D and B) as well as those of outliers is indicated. The lower panel shows the distribution of the GC content along the chromosome.

We next quantified differential transcript abundance for chromosomal genes. The expression of inactive gyrase alone modified significantly (Padj < 0.05) the expression of 143 genes (fold change > 1.25) most of which (80%) were downregulated in agreement with the hypothesis that the DNA binding activity of gyrase alone impedes transcription (Figure 4 A). This suggested a dominant negative effect that could be explained by the formation of a stable complex in which DNA is wrapped around the CDT domain of the gyrase heterotetramer. Without the possibility to cut the DNA, it is possible that the catalytically dead gyrase would stay bound to DNA thus forming a mechanical barrier for the passage of the RNA polymerase. When active gyrase is expressed, 410 genes were affected which corresponds to ∼18% of total number of annotated genes. Finally, by comparing TKgyrAB and TKY119F expression profiles we identified 205 DEGs which specifically responded to negative supercoiling activity of the gyrase. The comparison of the relative transcript abundances for these genes using *Z*-score scaling shows an opposite tendency between the gyrase expressing strain and the two control strains (Supplementary figure S6). We have therefore conducted further analyses on this set of genes which we named SRGs for supercoiling-responding genes.

SRGs were distributed throughout the entire chromosome of *T. kodakarensis* without obvious bias with respect to GC skew or transcription direction (Figure 4, B). We did not detect SRGs-specific GC content in the promoter region of SRGs or bias in the size of the corresponding transcripts (Supplementary figure S9). We noticed however, hotspots of deregulated genes in vicinity of ribosomal RNA genes. These regions would be expected to be targeted by the gyrase because the flanking regions of heavily transcribed ribosomal RNA genes are rich in supercoiled DNA (65).

The most highly deregulated SRGs encode the archaellum components (FC ∼ 54) and the genes involved in chemotaxis which are both highly upregulated (FC ∼ 6). An opposite downregulation trend was observed for genes assigned to energy production and conversion, nucleotide transport and metabolism and cell cycle control, cell division and chromosome partitioning functional categories (Supplementary figure S7). Among 11 DNA-repair related genes (66) only TK0784 encoding a homologue of XPD was significantly deregulated (log_2_FC = 0,33) suggesting that gyrase expression does not induce high levels of DNA damage. Intriguingly, however, the genes encoding Topo VI or Topo III were not deregulated although these enzymes are known to relax negatively supercoiled DNA *in vitro* (Supplementary Table S5). A notable exception was the reverse gyrase-encoding gene showing significant upregulation (*P*_adj_ = 0.007) albeit with a low fold change (FC = 1.2). We next looked into the transcript level of gyrase and reverse gyrase which, although an imperfect proxy of protein quantity, can give some insight into relative protein abundances in cells. To be able to compare the read counts within each sample we converted them into fragments per kilobase million (FPKM, Supplementary table S6). Assuming that all the transcripts are translated into functional protein, this shows that reverse gyrase, Topo VI and Mini-A (distant homolog of type IIB topoisomerases, (67)) are present in comparable levels in *T. kodakaransis*. In GyrAB strain average FPKM values are 1.5 for GyrA, 0.6 for GyrB and 0.47 for reverse gyrase suggesting that the gyrase is the most abundant topoisomerase in the GyrAB strain. From these data, we can speculate that the observed upregulation of reverse gyrase in the GyrAB strain was insufficient to counteract the gyrase negative supercoiling activity as suggested by the topology of the plasmids.

The above results demonstrated that gyrase introduction in *T. kodakarensis* provoked a genome-wide but, in most cases, mild deregulation of gene expression. The data also suggested that the gyrase-derived negative supercoiling was not handled by the endogenous topoisomerases.

**Figure 5.**
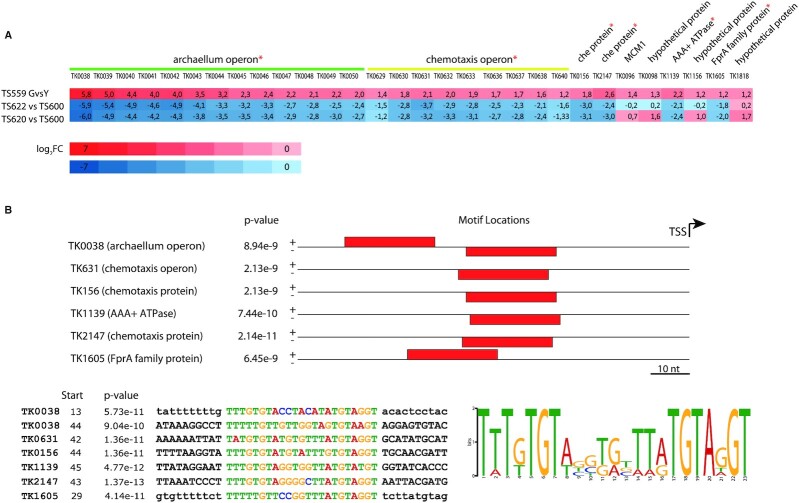
Top upregulated SRGs carry specific sequence motif in their promoter region. (**A**) Differentially expressed genes reacting to gyrase supercoiling activity or chromatin defect. Rectangles correspond to the top 30 upregulated SRGs and their colour corresponds to the log_2_FC values as indicated by the scale. The log_2_FC values of the genes/operons indicated by an asterisk are systematically anti-correlated between the gyrase expressing strain and the *T. kodakarensis* TS620 and TS622 strains. (**B**) Upper panel shows that the anti-correlated genes/operons invariably contain a conserved 23 bp sequence motif, indicated as red rectangle, upstream of the transcription start site (TSS). The lower panel shows the alignment of the 23 bp sequences on the left and the corresponding sequence logo on the right.

To understand better the molecular bases underpinning the observed transcriptional response, we compared our data with previously published analyses of *T. kodakarensis* transcriptomes. Among six available datasets one caught our attention as, similar to our study, the *fla* (archaellum) and *che* (chemotaxis) operons were the most highly deregulated (68). Sanders and colleagues studied the transcriptional profile of *T. kodakarensis* that either could not build multimeric chromatin particles (strain TS620, ΔHTkB HTkA^G17D^) or relied only on Histone B for building histone polymers (strain TS622, HTkB^WT^ HTkA^G17D^). Interestingly, out of top 30 upregulated SRGs, at least 26 were downregulated in strains TS620 and TS622 (Figure 5A). Most of these genes belonged to *fla* and *che* operons but we also identified two non-operonic chemotaxis genes (TK0156 and TK2147), one gene annotated as AAA + ATPase (TK1139) and a predicted FprA family A-type flavoprotein electron transfer protein (TK1605). When we extended this analysis to all SRGs (298 genes with *P*_adj_ < 0.05) we did not find any correlation with transcriptomes of TS620 and TS622 (Supplementary figure S8). Together, these data suggested that only the most highly upregulated SRGs are sensitive to both gyrase induced negative supercoiling and chromatin structural defect. To further examine the molecular basis for such behaviour we mapped transcriptional start sites (TSS) for anti-correlated SRGs based on experimental data from Jäger and coll. (69). The six identified intergenic sequences were subjected to MEME analysis (70) which revealed the presence of a 23 bp common motif (consensus sequence TTTGTGTABSTGBTTATGTAGGT) present in one copy or in two copies for *fla* operon. The motif, mostly located ∼35 bp upstream of TSS, does not resemble consensus promoter motifs of *T. kodakarensis* which typically have a B recognition element (BRE) followed by a TATA-box ∼33 and ∼23 bp, respectively, upstream from transcription initiation (69) (Figure 5B). A search for the motif in whole *T. kodakarensis* genome using FIMO (70) retrieved the six already identified motifs but no additional high scoring hits.

This analysis thus uncovered a common sequence motif found exclusively in the promoter region of the most highly upregulated SRGs, and further suggests that the dysregulation of these genes might be the consequence of alterations of chromatin structure induced by the negative-supercoiling activity of the gyrase.

### Impact of gyrase on *Thermococcus kodakarensis* growth

**Figure 6.**
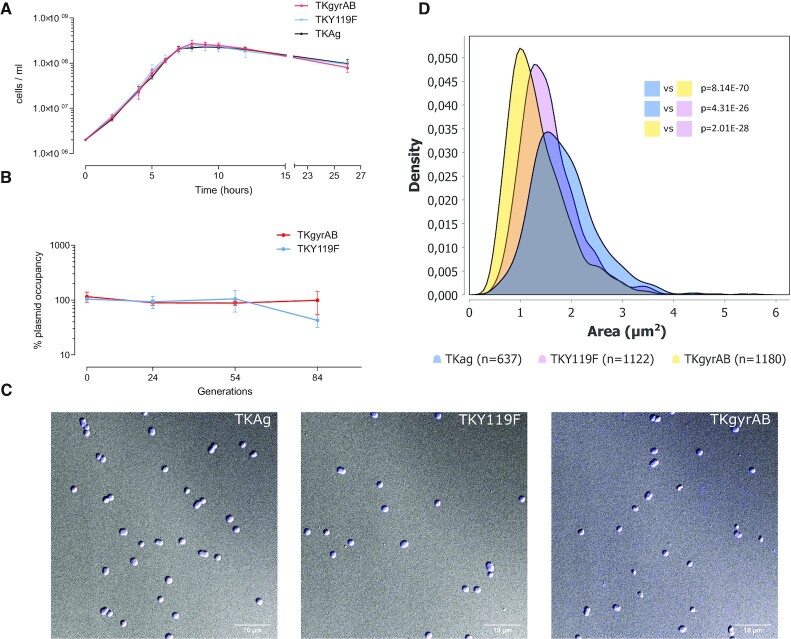
Expression of active DNA gyrase is not toxic for *T. kodakarensis*. (**A**) Growth of the three strains was monitored by direct cell counting using a Thoma cell counting chamber. The specific growth rates (number of divisions h^−1^) of the control (TKY119, TKAg) and gyrase expressing strain TKgyrAB) were 2.11 ± 0.27 h^–1^ TKAg, 1.45 ± 0.35 h^–1^ TKY119 and 1.81 ± 0.37 h^–1^ TKgyrAB. Specific growth rates were calculated from the slope of the linear portions of the curves according to the equation μ = d*Y*/d*t*, where *t* is time and *Y* is the cell density. Mean values of *Y* were used for the calculation. Error bars represent the standard deviation, *n* = 3. The differences between the slopes are not significant, *P* = 0.34, ANCOVA two-tailed test. (**B**) Measurement of plasmid loss over 84 generations corresponding to 14 subcultures. Each point corresponds to the ratio of CFUs grown on plates under non-selective or selective medium. The experiment was done in triplicate and the bars correspond to standard deviation from the mean value. (**C**) Representative micrographs of *T. kodakaransis* cells harvested at the exponential growth stage. The cells were stained with Hoechst dye and DIC and fluorescent images were superposed. (**D**) Density plot of cell area determined from DIC microscopy images. The number of analysed cells is indicated below the graph.

To assess the impact of gyrase on *T. kodakarensis* growth we first monitored the growth kinetics of the three strains in batch cultures and at optimal growth conditions by direct cell counting. The recorded growth curves exhibited a typical sigmoidal shape and were overall similar (Figure 6A). The specific growth rate at the exponential phase was lower for TKgyrAB and TKY119F as compared to TKAg control strain, however, the slopes were not significantly different (*P* = 0.34) indicating that the observed differences are not significant.

Next, the cell shape and size as well as DNA content were assessed using light microscopy coupled with image analysis. This revealed that the exponential phase TKgyrAB cells had the typical irregular round shape as the control cells and contained DNA. However, the measurement of cell area revealed that the gyrase expressing strains were, on average, smaller than the TKAg strain and this difference was statistically significant (*P* = 8.1 × 10^–70^, Figure 6B, Supplementary figure S10). The phenotype was the most pronounced for TKGyrAB cells which (assuming that the cells are perfectly spherical) exhibited in average 35% less volume compared to TkAg control. Despite being smaller, the TKGyrAB cells did not exhibit a filamentous phenotype indicating they divided at a normal rate (Supplementary Figure S10).

We next tested whether the gyrase expression becomes toxic during longer culturing. If so, we expected that the gyrase expression plasmid would be gradually lost in non-selective culture conditions. We therefore quantified the gyrase expressing plasmid in the population by counting colony forming units (CFU) under selective versus non-selective conditions over 84 generations (14 subcultures). Plasmid was stably maintained in the TKgyrAB strain while about 50% plasmid loss was observed for strain TKY119F at the final stage of the experiment (Figure 6C). To ensure that the experiment was performed with cells that contained active gyrase we monitored pTPTK2 topology using 1D agarose gel electrophoresis (Supplementary figure S11). The amount of the extreme supercoiled form of pTPTK2 remained constant throughout the experiment indicating no loss of gyrase activity during prolonged culturing of *Thermococcus*.

Collectively, the data show that the gyrase is remarkably well tolerated by *T. kodakarensis*.

## DISCUSSION

In this study, we demonstrate that it is possible to introduce active bacterial gyrase in the hyperthermophilic archaeon *T. kodakarensis*, a cellular system that naturally lacks negative supercoiling activity. We further show that, as in bacteria, the gyrase became the dominant topoisomerase converting positively supercoiled plasmids into highly negatively supercoiled DNA. Transcriptomic analyses revealed mild deregulation of hundreds of genes including induction of stress – related flagellar (archaellum) and chemotaxis systems. The analysis of the top 30 upregulated genes (including *fla* and *che* operons) revealed the presence of a conserved 23 bp sequence motif in their promoter region. These genes were also systematically downregulated in *T. kodakarensis* strains carrying mutations in genes encoding histones. Reverse gyrase was the only topoisomerase of *T. kodakarensis* for which the expression was altered (slightly upregulated) in response to the negative supercoiling activity of gyrase. Despite global-scale alterations of its cellular context, the *T. kodakarensis* growth rate was not affected, suggesting that critical DNA-templated processes were not compromised by gyrase activity.

This was an unexpected result because the negative supercoiling activity of the gyrase should, in principle, interfere with essential processes such as transcription or DNA replication. In particular, gyrase relaxes positive supercoils accumulating ahead of transcribing RNA polymerase (18), a task predicted to be accomplished by Topo VI in archaea (71). Moreover, negative supercoiling facilitates DNA melting which in turn facilitates promoter firing (64) but, at high temperatures, also exposes ssDNA to heat-induced damage (72,73). In spite of these potential threats to genome stability and expression, *T. kodakarensis* expressing gyrase tolerated an approximately five-fold increase in the negative supercoiling density of plasmid DNA as compared to the natural plasmid DNA topology. Granted, plasmid DNA topology does not necessarily recapitulate chromosomal DNA topology, this observation is still quite impressive when put in perspective with the life-limiting tolerance level of bacteria. *Streptococcus pneumoniae* can only tolerate increases in negative supercoiling levels of up to 1.4-fold (≈41% increase for plasmid DNA) (74) and *E. coli* of up to 1.1 fold (≈14% increase for plasmid and chromosomal DNA) (75). Besides, the RNA-seq data do not show notable induction of genes involved in DNA repair thus suggesting that the gyrase expression does not result in massive DNA damage in *T. kodakarensis*.

Why is gyrase so well tolerated by an organism in which the cellular DNA and machineries did not co-evolve to accommodate pervasive negative supercoiling activity? We suggest that during evolution, and in *T. kodakarensis*, the wrapping of genomic DNA in nucleosome-like structures may have facilitated the establishment of the gyrase in archaeal cells. In Euryarchaea, histones have evolved as most abundant chromatin proteins (76,77) and *T. kodakarensis* has particularly dense histone coverage approaching 100% (69,78–80). Commonly, archaeal histone dimers assemble into tetramers as minimal nucleosomal units that wrap ∼ 60 bp of DNA (81) but in some archaea, including *T. kodakarensis*, the tetramers can be extended via incorporation of additional dimers into particles of variable size that wrap up to 480 bp of DNA in negatively constrained supercoils (78,82,83). This flexible chromatin structure may participate in adaptive responses of *T. kodakarensis* by restructuring the existing pool of histones such that the local gyrase-induced increase of negative supercoiling would be efficiently absorbed. In line with this idea, we discovered that the most highly upregulated SRGs are almost systematically downregulated in *T. kodakarensis* strains carrying mutations in histone genes. Remarkably, the anti-correlated transcriptional response is always associated with a 23 bp motif occurring about 35 bp upstream of transcription start site of these SRGs. The function of this motif is currently unclear but we can speculate that its presence may render the promoter region particularly sensitive to alterations of DNA topology. Along the same line, it is interesting to note that artificial chromatinization of the *E. coli* genome with archaeal histones resulted in downregulation of gyrase genes as a part of the adaptive cellular response (84). Together, these observations gathered in artificial settings suggest that both organisms tolerate well the introduction of major modellers of DNA topology and react by balancing chromatin structure and DNA supercoiling to achieve a DNA geometry necessary to sustain life.

Once established, the gyrase became fixed in many archaeal lineages to the point where it has become an essential protein in present-day archaea. Indeed, early studies showed that gyrase-targeting drugs such as novobiocin and ciprofloxacin inhibited growth of different archaea including methanogens, halophiles and thermoacidophiles (85). These experiments also established that gyrase was responsible for introducing most, if not all of the negative supercoils in plasmid DNA molecules (85,86). Notably, novobiocin treatment of *Halobacterium halobium* cultures stopped DNA replication specifically and instantaneously indicating, albeit indirectly, that gyrase acts upon chromosomal DNA during the elongation step of DNA replication (85). Collectively, these findings suggest that the negative supercoiling activity of the gyrase was positively selected in the course of evolution, but the selective advantage conferred by this feature remains to be established and future studies of *in vivo* functions of archaeal gyrases should bring some insight. In Bacteria, gyrase-controlled negative supercoiling is instrumental for quick adaptation of the cellular protein repertoire to changing environmental conditions (20) but whether such mechanisms operate in gyrase-encoding Archaea is not known. Indirect evidence points to involvement of negative DNA supercoiling in gene expression control in extreme halophiles. In these organisms, a plasmid-encoded *gyrB* gene and chromosomally encoded *bop* gene (encoding bacteriorodopsin) were strongly induced (up to 20-fold) by DNA relaxation in novobiocin-treated cultures, a drug that inhibits gyrase activity (87,88). More recently, a global transcriptome analysis was reported for novobiocin-treated *Halobacterium* species (89). The expression of many genes was affected including the upregulation of gyrase, topoisomerase VI and topoisomerase I expression indicating the involvement of these enzymes in regulation of the DNA supercoiling levels in this organism (89). However, to what extent the gyrase controls the chromosomal supercoiling and how its activity is coordinated with other archaeal topoisomerases, histones and NAPs is currently unknown.

Related to that, it is noteworthy that reverse gyrase was the only topoisomerase of *T. kodakarensis* that responded to the negative supercoiling activity of the gyrase. Reverse gyrase is a Topo I enzyme and the only topoisomerase capable of supercoiling DNA positively (32). Through this activity, reverse gyrase can remove negative supercoiling (32,33,90,91). Remarkably, reverse gyrase is found specifically in thermophilic organisms and its deletion in *T. kodakarensis* and *Pyrococcus furiosus* is lethal at 93 and 95°C, respectively (34,35). It was initially suggested that reverse gyrase prevents thermal denaturation of the double helix by introducing positive supercoils in chromosomal DNA (32–34). However, the idea that positively supercoiled DNA, in spite of its stabilising effect, is not essential for a hyperthermophilic lifestyle was put forward some time ago based on the observation that thermophilic *Thermotoga* bacteria and gyrase-encoding hyperthermophilic Archaeoglobi archaea contain negatively supercoiled plasmids (40,58). Later studies reported the involvement of this topoisomerase in DNA repair (92–95). Recently, however, a study of gyrase-less *Saccharolobus* (formerly *Sulfolobus*) *solfataricus* reported that reverse gyrase is involved in homeostatic control of DNA supercoiling mainly based on the finding that the protein abundance increased about two-fold when cells were exposed to supraoptimal temperatures and the enzyme was more active *in vitro* (91). Our data show that 1.2-fold (at the mRNA level) upregulation of reverse gyrase in *T. kodakarensis* is insufficient to restore natural DNA topology and suggest that, at least in *Thermococcus* and in these artificial conditions, this topoisomerase is not essential for homeostatic control of DNA topology.

In contrast, we were intrigued to find that the transcription profile of plasmid-encoded genes in the gyrase-expressing *T. kodakarensis* seems not to be modified. The most clearly identified effect of supercoiling on transcription initiation results from the requirement of RNA polymerase to open the double helix in order to gain access to the template strand. Negative supercoiling facilitates melting of the double helix and a strong regulatory effect of negative supercoiling upon gene transcription is well recorded in bacteria (21,65). In hyperthermophiles, which have relaxed or slightly positively supercoiled DNA, it is thought that the elevated growth temperature may replace negative supercoiling as source of melting energy (96,97) and we therefore expected that the gyrase–induced negative supercoiling and high temperature would synergistically activate the expression of plasmid-borne genes in *T. kodakarensis*. The fact that this is not so suggests that *T. kodakarensis* is naturally equipped to allow transcription from topologically different DNA templates. This is reminiscent of observations made by Bell and colleagues who studied the effect of temperature and template topology on expression from an archaeal ribosomal RNA promoter using a highly purified *in vitro* system from crenarchaeon *Sulfolobus* (63). They found that, in marked contrast to characterised bacterial and eukaryal systems, DNA template topology had negligible effect on transcription levels at 78°C, the optimal growth temperature for *Sulfolobus*. In another study, Hethke *et al.* used the cell-free transcription system of *Pyrococcus furiosus*, a euryarchaeon closely related to *Thermococcus*, to study the effect of DNA template topology on expression from the *gdh* promoter at 70 and 90°C (73). They found that at both temperatures negatively supercoiled DNA was the preferred template compared with relaxed DNA and that positive supercoiling deteriorates the template activity of DNA. It is unclear whether the differences between *P. furiosus* and *Sulfolobus* transcription systems come from the type of promoter used or from the intrinsic properties of their transcription machineries (73). We can now use the gyrase-expressing *T. kodakarensis* to perform similar studies *in vivo* and thus study the relationship between supercoiling and gene expression in more natural settings.

Would global negative supercoiling be advantageous to a hyperthermophile at lower, suboptimal temperatures? The experiments described by Bell and colleagues suggest so since the *Sulfolobus* system was unable to transcribe relaxed or positively supercoiled templates at 48°C. They further highlighted that, in response to cold shock, hyperthermophiles rapidly reduce their plasmid linking number (48,98) raising the possibility that global regulation of DNA superhelical state *in vivo* represents an effective mechanism for ensuring continued gene expression after drastic changes in temperature of the environment. Building upon these findings, more than 20 years ago, P. López-García proposed that archaea may have improved their adaptability to mesophily by importing the gyrase from bacteria (99). This idea was reinforced later on by the finding that the vast majority of gyrase-encoding monophyletic group II Euryarchaea are mesophiles in spite of their thermophilic origin (13,26,28). These archaea possess histones leading to the proposal that the acquisition of the gyrase may have had a synergistic effect on DNA-dependent processes in these organisms, with associated changes in transcriptional patterns thus contributing to bacterial-like progressive adaptation to lower temperatures (28). The gyrase-expressing *T. kodakarensis* now offers the possibility to test this evolutionary hypothesis and ultimately understand why several archaeal lineages became addicted to gyrase.

## DATA AVAILABILITY

The RNA-seq data reported in this article are available in ArrayExpress (https://www.ebi.ac.uk/arrayexpress/) database and can be accessed with E-MTAB-10799 accession number. Processed tables, which were modified to homogenize gene names, are available upon request.

## Supplementary Material

gkab869_Supplemental_File
