# Supplementary material for: The hyperthermophilic archaeon Thermococcus kodakarensis is resistant to pervasive negative supercoiling activity of DNA gyrase
Source: Nucleic Acids Res. 2021 Nov 10;49(21):12332–47. doi: 10.1093/nar/gkab869 (PMC8643681; doi:10.1093/nar/gkab869)
Supplement: gkab869_Supplemental_File [file gkab869_supplemental_file.pdf]

SUPPLEMENTARY FIGURES AND TABLES

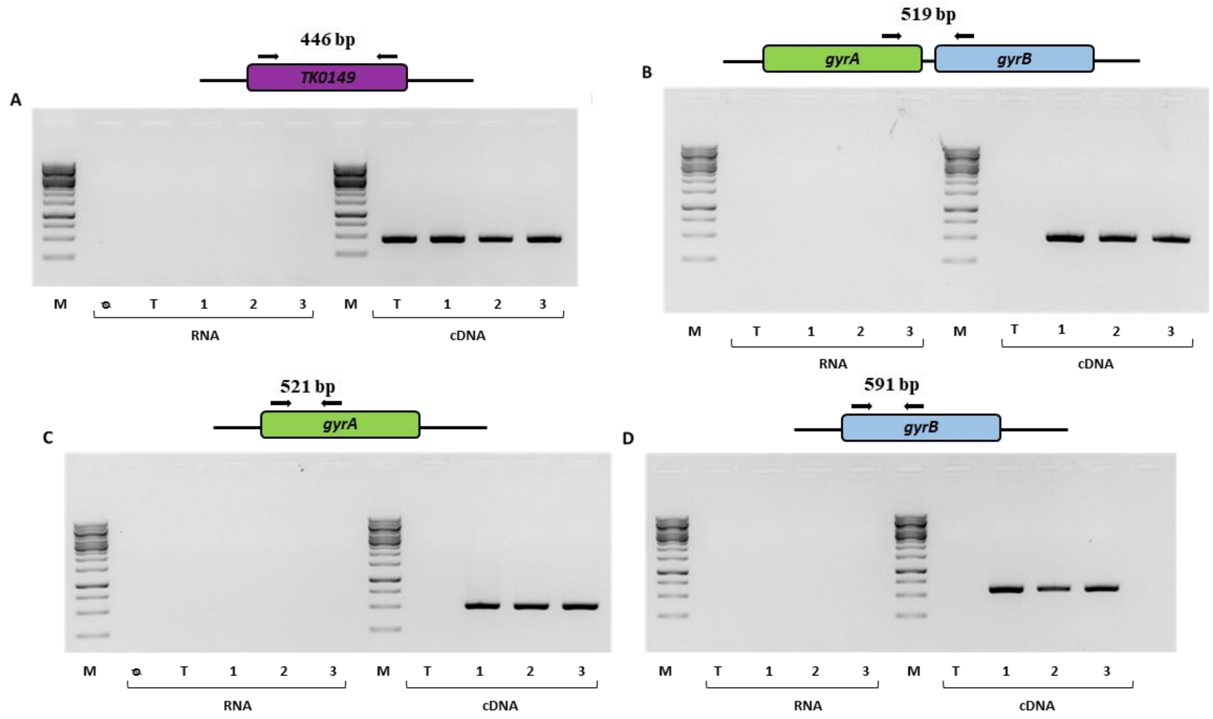

Supplementary figure 1. The *gyrA* and *gyrB* from *T. maritima* are transcribed in *T. kodakarensis* TKgyrAB

The expression of *gyrA* and *gyrB* was analysed by RT-PCR for three independent clones of strain TKgyrAB. For each clone, total RNA was isolated and then retrotranscribed to cDNA (see material and methods). The total RNA (left side of the gels) or cDNA (right side of the gels) was used as template for PCR and the obtained products were separated by agarose gel electrophoresis. The position of the used specific oligonucleotides is indicated with arrows. T is a control PCR where total RNA or cDNA from *T. kodakarensis* TKAg which carries the empty expression vector was used as template. TK0149 is plasmid-encoded pyruvoyl-dependent arginine decarboxylase which confers prototrophy to agmatine.

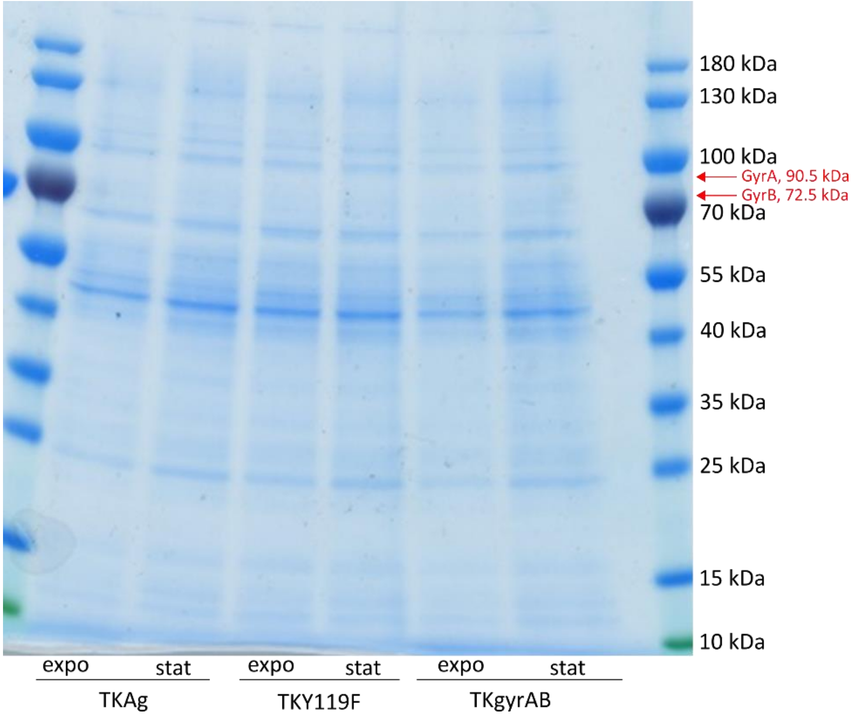

Supplementary figure 2. SDS-PAGE analysis of soluble proteins from *T. kodakarensis*.

TKAg, TKY119F and TKgyrAB were grown until exponential (expo) or stationary phase (stat). Cells were pelleted and resuspended in Laemmli buffer and heat-denatured. Resulting cell lysates were analysed on a 4-20% polyacrylamide gel. Protein ladder sizes are indicated on the right side of the gel. Expected bands at 90,5 kDa and 72,5 are not visible in TKY119F and TKgyrAB lysates indicating that *gyrA* and *gyrB* are not overexpressed.

A

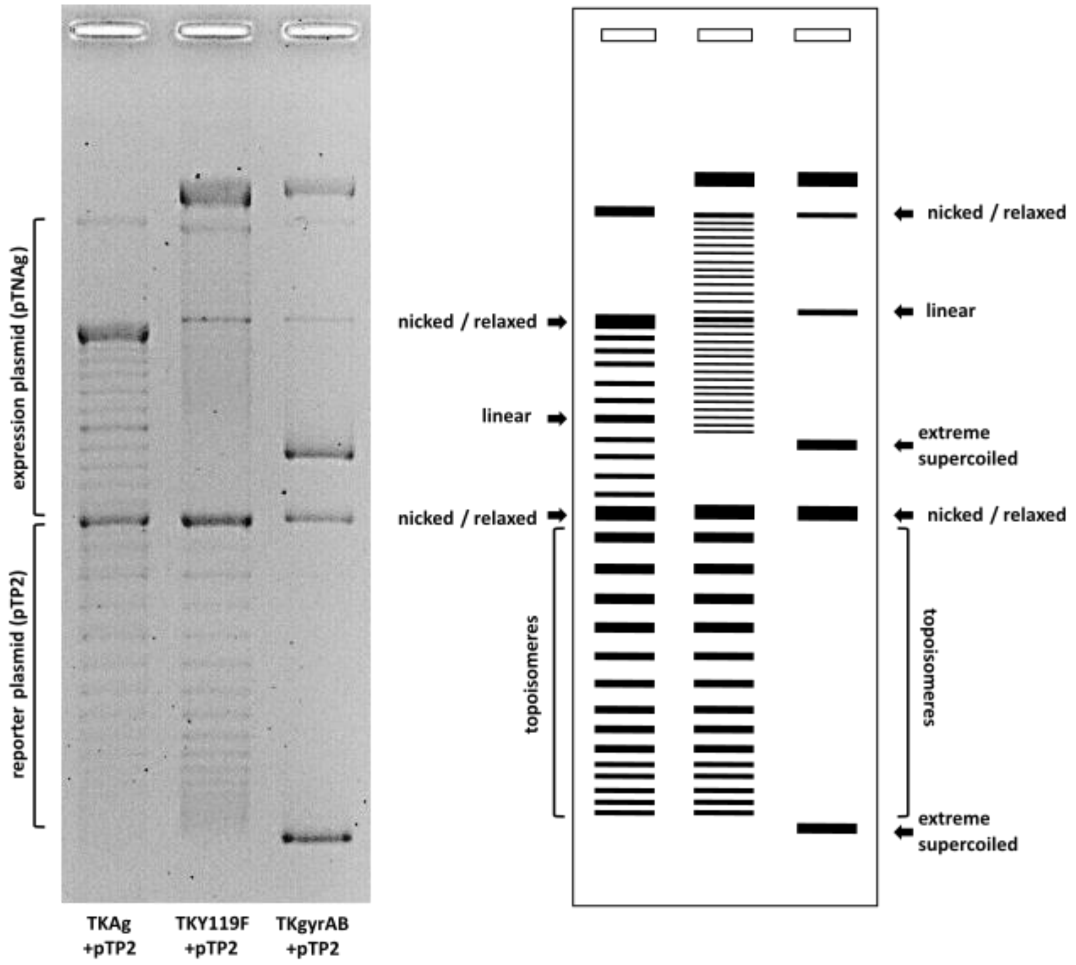
expression plasmid (pTNAg)
reporter plasmid (pTP2)
TKAg
+pTP2
TKY119F
+pTP2
TKgyrAB
+pTP2
nicked / relaxed
linear
nicked / relaxed
topoisomeres
nicked / relaxed
linear
extreme
supercoiled
nicked / relaxed
topoisomeres
extreme
supercoiled

B

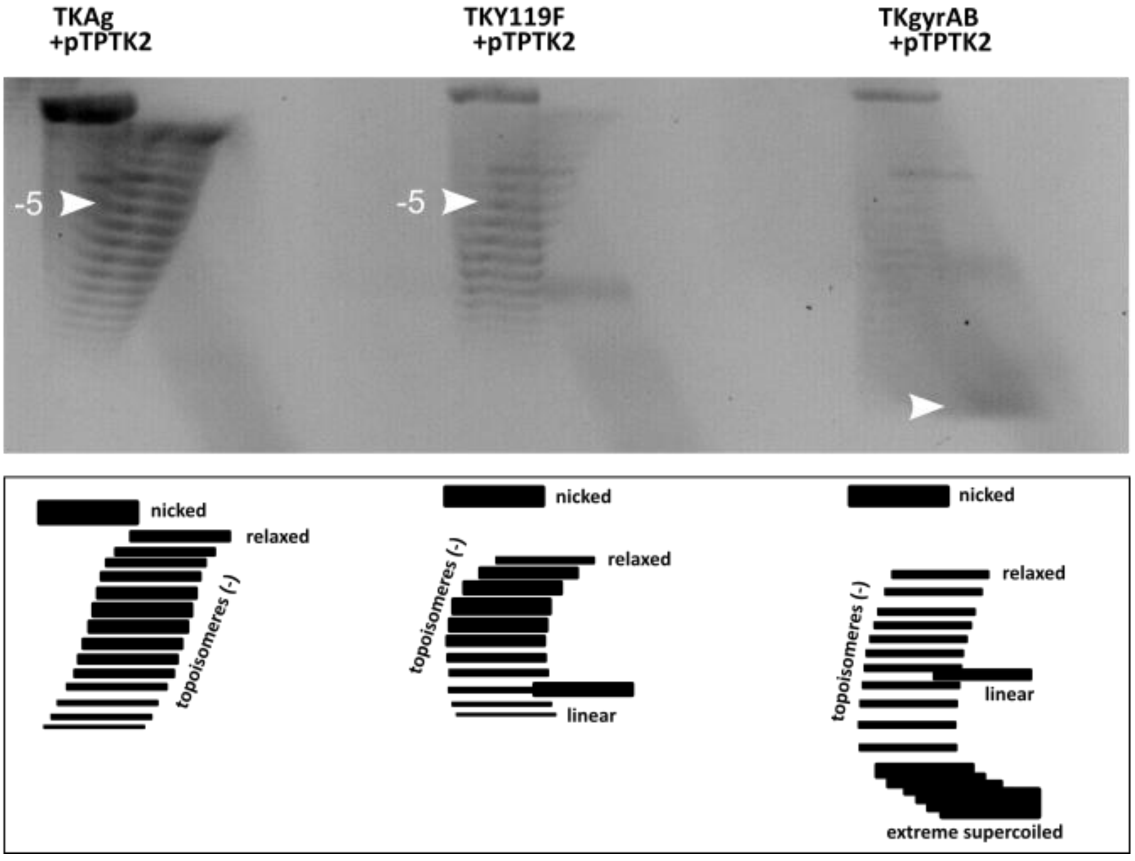
TKAg
+pTPTK2
TKY119F
+pTPTK2
TKgyrAB
+pTPTK2
-5
-5
nicked
relaxed
topoisomeres (-)
nicked
relaxed
topoisomeres (-)
linear
nicked
relaxed
topoisomeres (-)
linear
extreme supercoiled

Supplementary figure 3. Topological profile of plasmids isolated from *Thermococcus kodakarensis* TKgyrAB, TKAg or TKY119F strains.

A) The plasmid DNA was migrated in 0.8% (w/v) agarose gel in absence of intercalating agents for 24h at 1,6 V/cm at 4°C. The ethidium bromide gel is shown on the left and its schematic representation on the right. Different topological forms of plasmids are indicated.

B) The plasmid DNA was migrated in two dimensions in a 1% (w/v) agarose gel prepared with 1X TEP (36 mM Tris-HCl, 30 mM NaH_2PO_4, 1 mM EDTA, pH 7.8). Chloroquine was added at 10 µg/ml only in the second dimension. First dimension was run for 15 h at 1.2 V/cm and second dimension was run for 5 h at the same voltage. Observed topological forms are indicated in the corresponding cartoon. For the sake of clarity only the part of the gel containing the reporter plasmid pTPTK2 is shown. The major topoisomer in TKAg and TKY119F strains is indicated with white arrow.

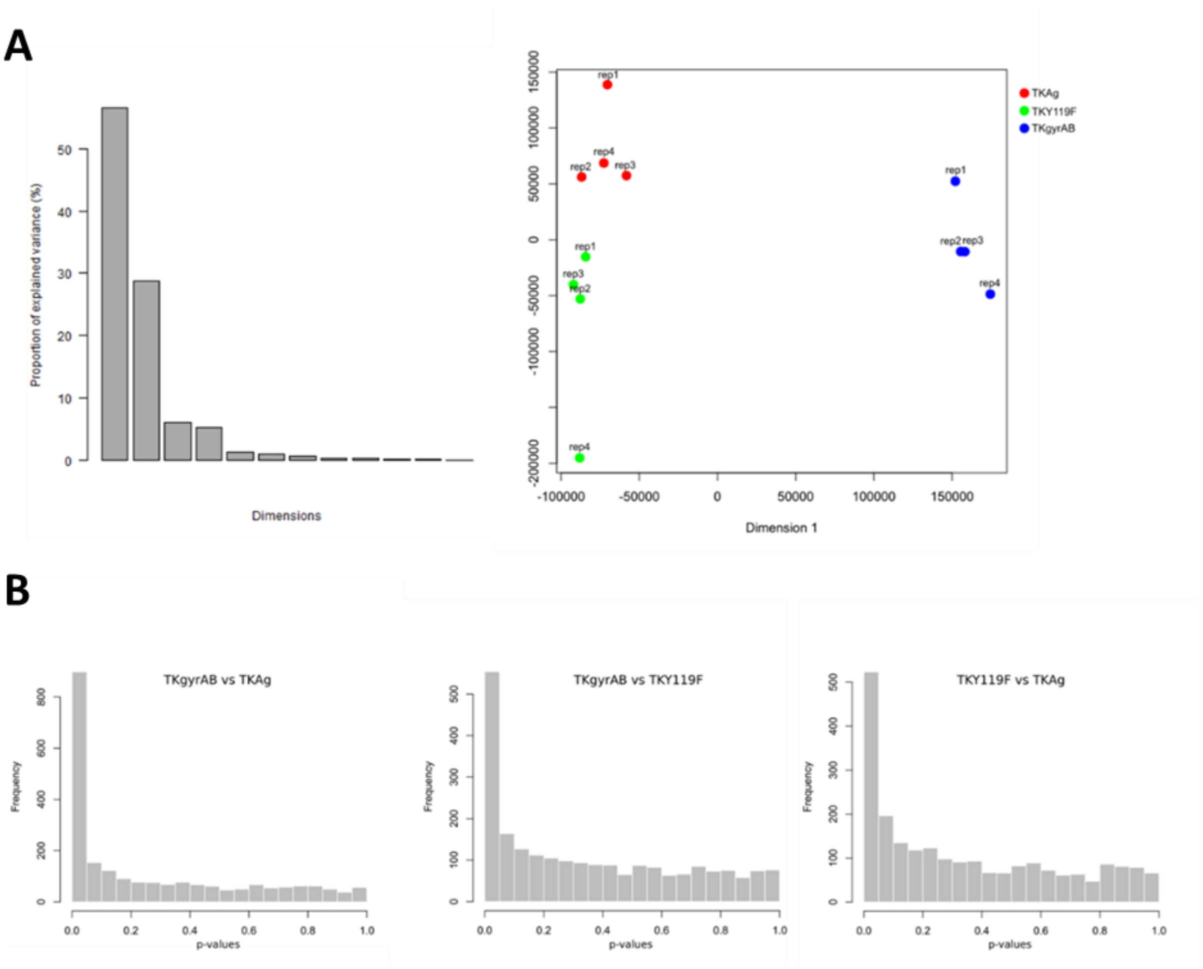

Supplementary figure 4: Quality assessment of RNA-seq data

A) Principal component analysis was performed to assess the variability in the dataset. Left graph shows the distribution of components as a function of data variability. The first two principal components capture more than 80% of the variance. In the graph on the right, each point corresponds to a single RNA-seq dataset. For each condition (strain) four biological replicates were analysed.

B) P-value histogram for the three differential analyses to evaluate the p-value significance threshold. The three plots show anti-conservative p-values distribution with the null p-values uniformly distributed between 0 and 1. Such distribution indicates that the frequency of the false positives in the three datasets will be low for the p-values less than 0.05.

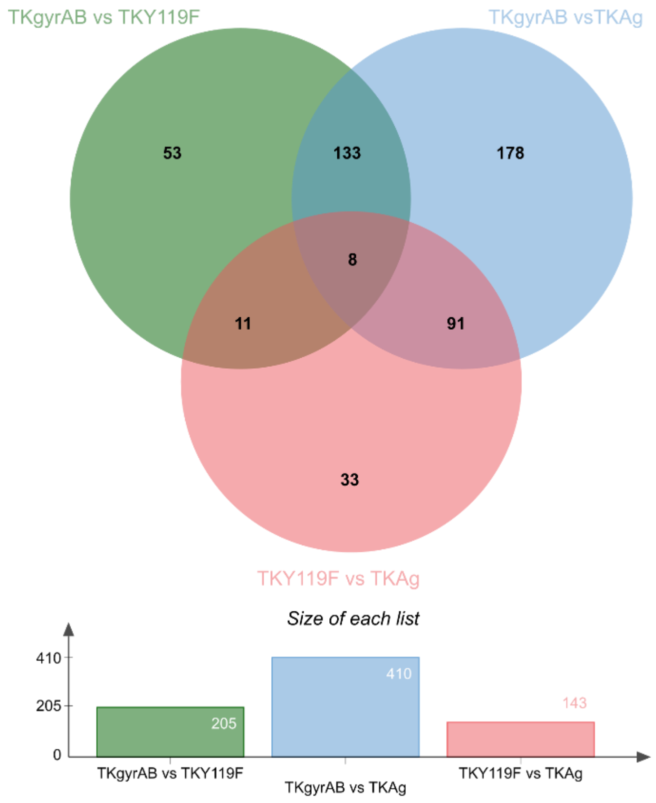

Supplementary figure 5: Venn diagram showing the overlap between the DEGs detected in three pairwise comparisons of transcriptomes. Green circle: genes that react to negative supercoiling; Pink circle : genes that react to gyrase expression and DNA binding; Blue circle : genes that react to negative supercoiling, gyrase expression and DNA binding. While there is significant overlap between pink and blue as well as green and blue circles, only few genes are shared between green and pink circle. This indicates that the genes identified by TKGyrAB vs TKY119F comparison are enriched in genes responding specifically to negative supercoiling activity of the gyrase.

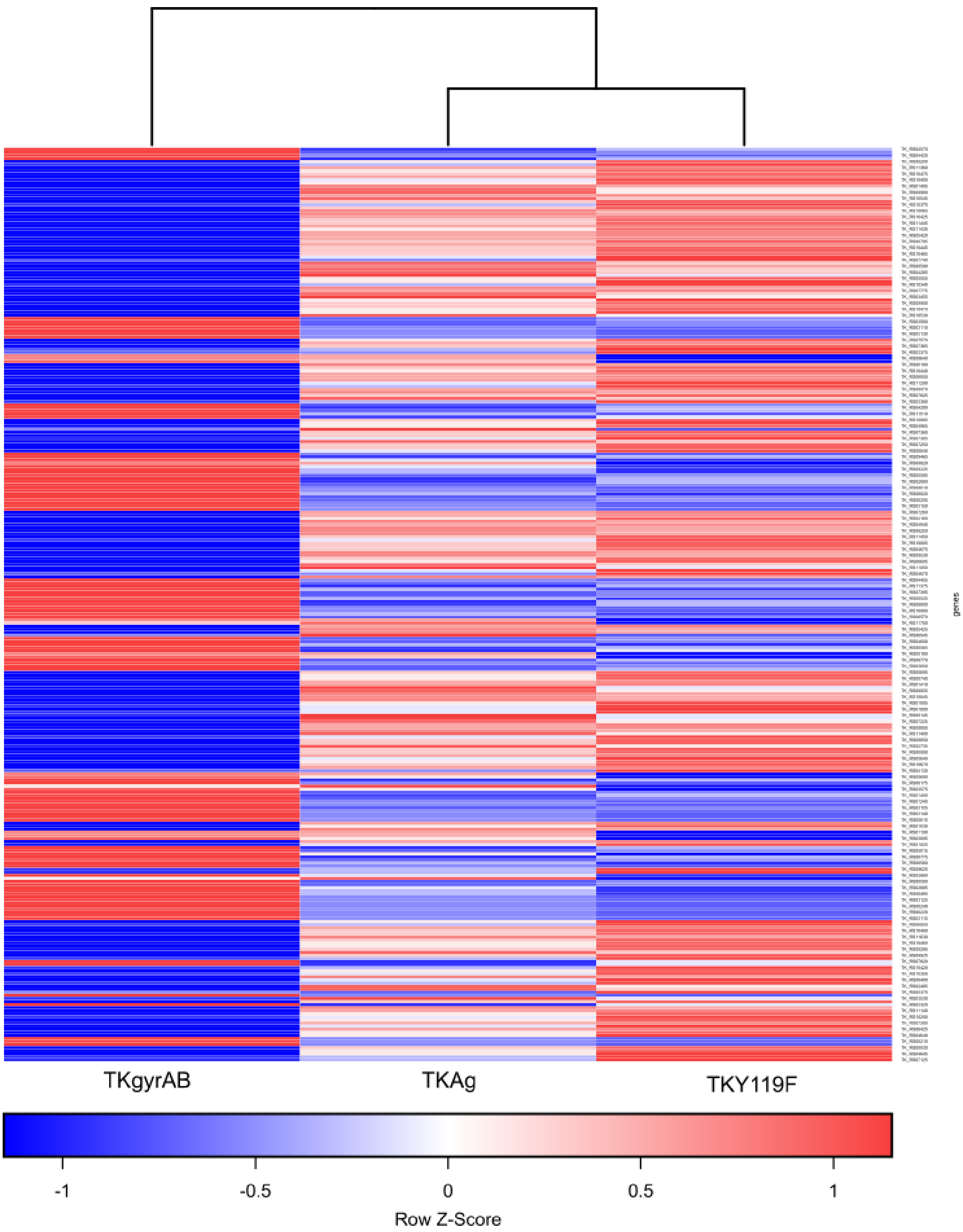

Supplementary figure 6. Relative count abundancies for supercoiling – responding subset of DEGs using Z-score scaling.

Each line corresponds to one gene and each column correspond to the one of the three recombinant strains as indicated below the heat map. The Z score for each gene is the mean value of four individual Z-scores obtained for each biological replicate. Individual Z-scores were calculated using normalised counts for each gene according to the standard equation. The two control strains (TKAg and TKY119F) are clustered together to exclusion of the gyrase expressing strain (TKGyrAB).

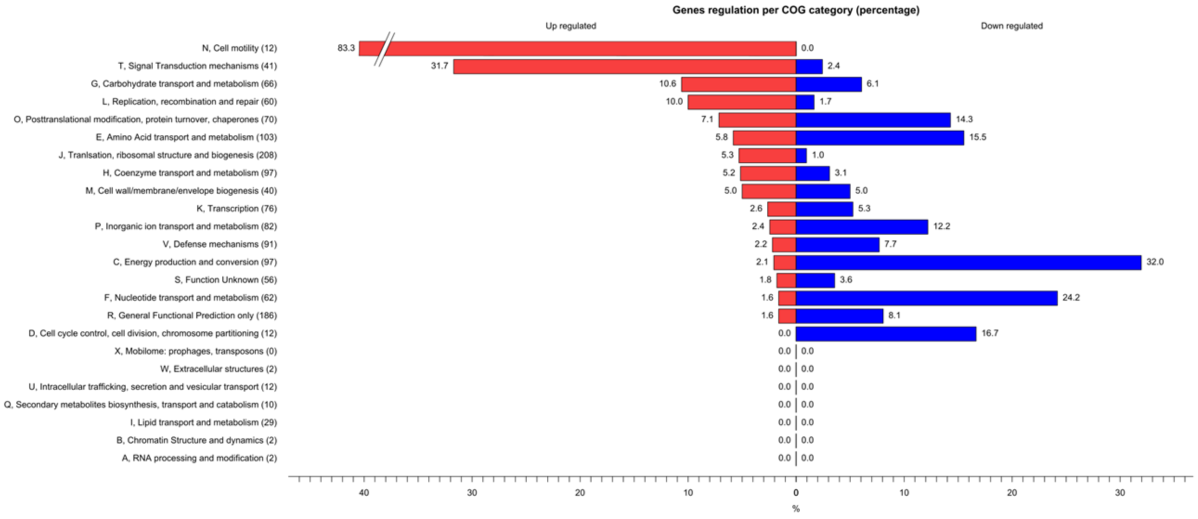

Supplementary figure 7: Assignment of DEGs to ArCOG functional categories.

Total number of genes assigned to each category is indicated in the brackets. The percentage of DEGs in each category is indicated next to each bar. Note that 1416 out of 2256 annotated protein coding genes of *T. kodakarensis* TS559 have been assigned functions.

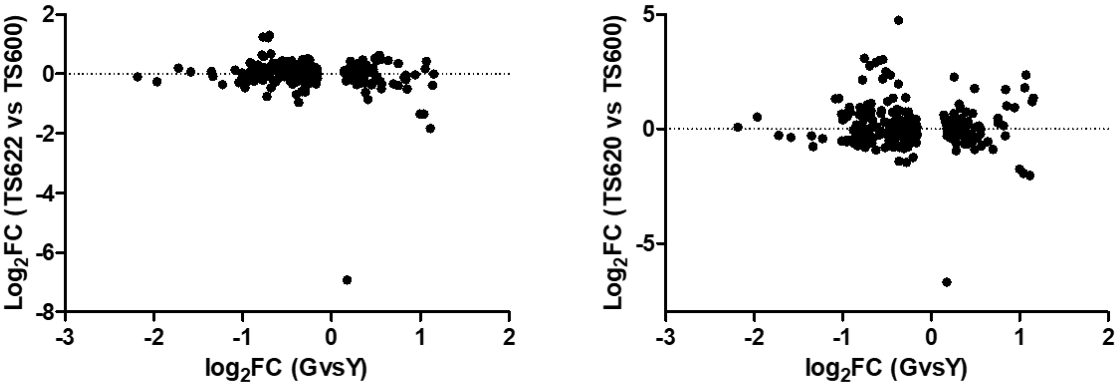

Supplementary figure 8: Correlation analysis of transcriptional output for genes responding both to supercoiling and chromatin defect.

Each dot corresponds to log_2FC values for SRGs with Padj < 0.05. The top 30 upregulated SRGs were removed from the analysis. Graph on the left shows the correlation with corresponding genes from *T. kodakarensis* TS622 (HTkB^{WT} HTkA^{G17D}) and on the left the correlation with TS620 (ΔHTkB HTkA^{G17D}). Non-parametric Spearman correlation test revealed the absence of significant correlation between the two sets of data.

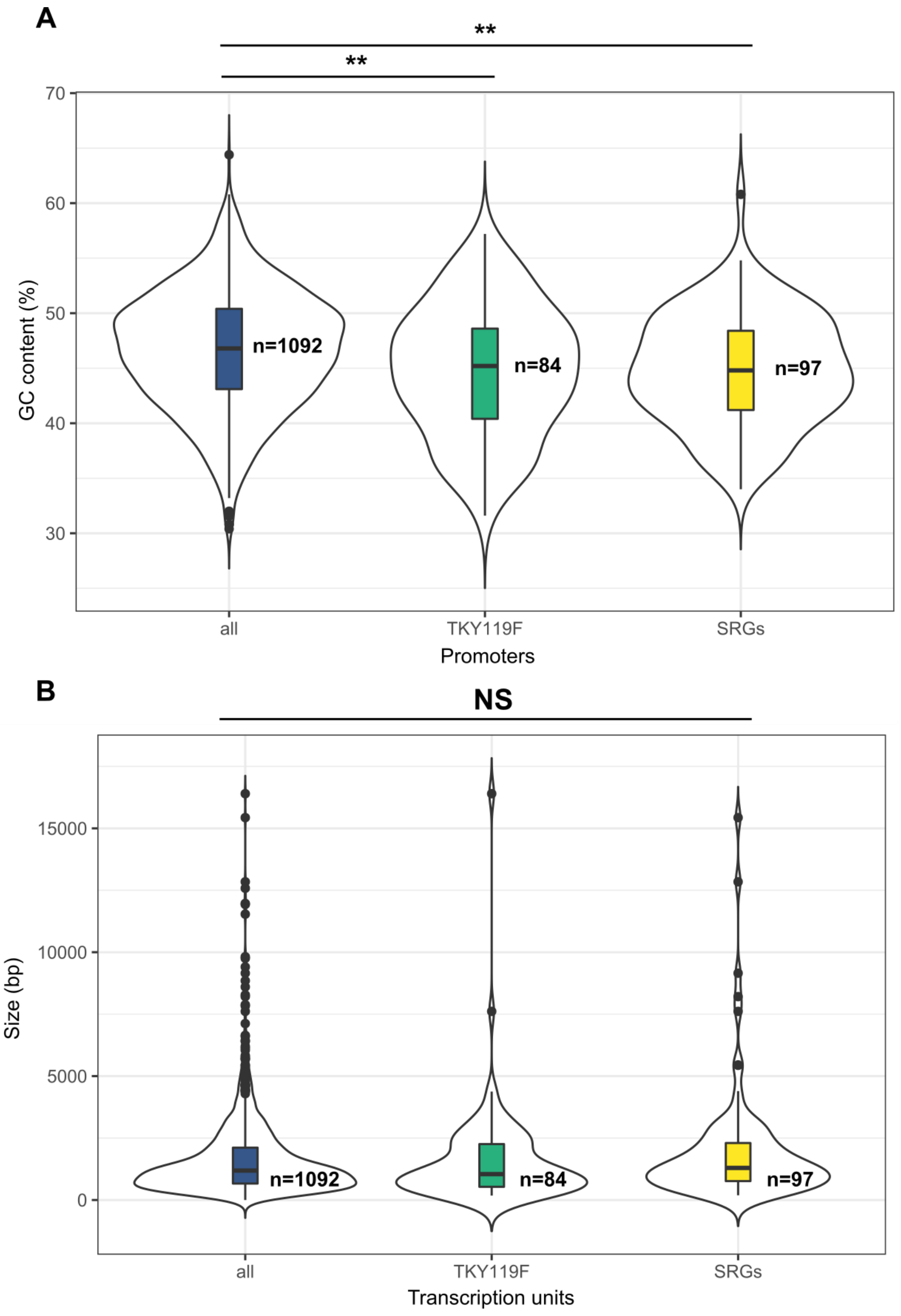

Supplementary figure 9: Analysis of GC content of SRGs promotor regions and length of the corresponding transcripts

Operons were predicted for *T. kodakarensis* TS559 genome using Operon-mapper software (1). The 250 bp upstream of each operon start and the predicted transcription unit size were extracted using R and the data were plotted using ggplot2 package.

A) GC content of promoter regions of SRGs (SRGs) were compared to all predicted promoter regions (all) in *T. kodakarensis* TS559 and to the promoter regions of the genes reacting to expression of the catalytic mutant (TKY119F). The median values were compared using Mann and Whitney non-parametric statistical test. Significantly different medians are indicated by ** (p value < 0.01) on the top of the graph.

(B) The median values of transcription units size analysis were compared by Kruskal-Wallis non-parametric statistical test. None of the medians tested is significantly different from the others (p value > 0.05).

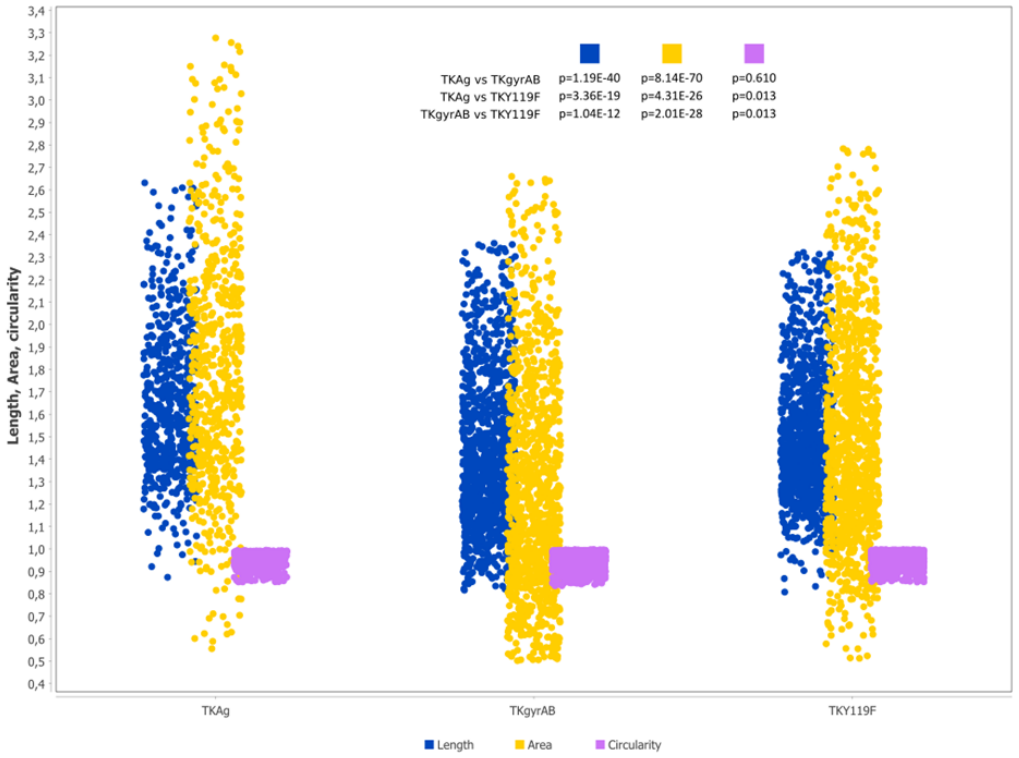

Supplementary figure 10. Analysis of cell size and shape of recombinant *T. kodakaresis* cells by DIC microscopy.

Box plots showing the distribution of the cell surface for the indicated number of cells as determined using MicrobeJ. The mean values were compared using nonparametric statistical test (Mann-Whitney U test) and the statistical significance is expressed as P values. Both TKY119F and TKGyrAB strains are significantly smaller than the control TKAg strain but the shape of TKGyrAB is not significantly different from the control strain.

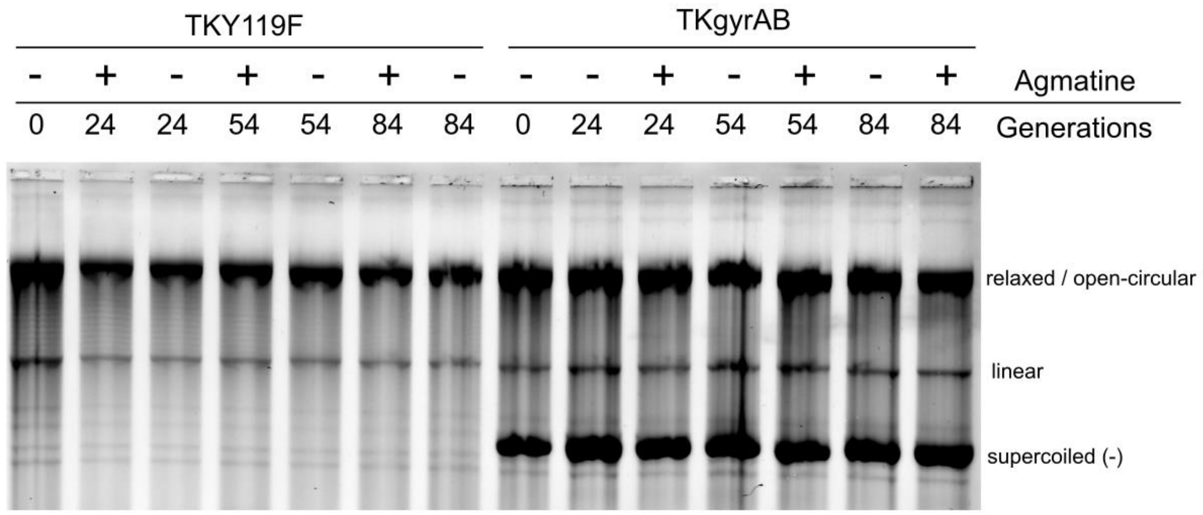

Supplementary figure 11. One dimensional gel agarose analysis of pTPTK2 topology over prolonged subculturing of *Thermococcus kodakarensis*.

pTPTK2 plasmids were isolated from the cultures after 24, 54 or 84 generations and separated on the 0.8% agarose gel in absence of chloroquine. The presence (non-selective condition) or absence (selective condition) of agmatine in culture medium is indicated above the gel.

Supplementary Table 1: Strains and plasmids used in this work

Strain name	Genotype	Markers	Source
Escherichia coli XL1-Blue	*endA*1 *gyrA*96 thi-1 *recA*1 *relA*1 lac *glnV*44 F'[::Tn10 *proAB*$^+$ *lacI*^{q} Δ(l*acZ*)M15] *hsdR*17	Tetracycline resistance<br>Nalidixic acid resistance	Stratagene
Thermococcus kodakarensis TS559	Δ*pyrF*; Δ*trpE::pyrF*, Δ*TK0664*, Δ*TK0149*	Uracil prototrophy<br>Tryptophan auxotrophy<br>Agmatine auxotrophy<br>6-methylpurine resistant	Santangelo 2010

Plasmid name	Genotype	*E. coli* marker(s)	*T. kodakarenesis* marker(s)	Source (Accession No.)
pTNAg	pLC70Δ(*TK0254-PF1848*):: (P_{TK0149}-*TK0149*)	AmpR, KanR	Agm	Catchpole 2018 (MG920813)
pLC70	see reference	AmpR, KanR	Trp, MevR	Santangelo 2008 (N/A)
pTPTK2	pTP2::(p15A-*cat*),(P_{TK2279}-*TK0254*)	CmR	Trp	Catchpole 2018 (MG920816)
pTNAg-gyrAB	pLC70Δ(*TK0254-PF1848*):: (P_{TK0149}-*TK0149*)	AmpR, KanR	Agm	*this work*
pTNAg-Y119F	pLC70Δ(*TK0254-PF1848*):: (P_{TK0149}-*TK0149*)	AmpR, KanR	Agm	*this work*

AmpR=ampicillin resistance; KanR=kanamycin resistance; CmR=chloramphenicol resistance; Trp=tryptophan prototrophy in a Δ*trpE* (TK0254) background; MevR=mevinolin resistance; Agm=agmatine prototrophy in a ΔTK0149 background.

Supplementary Table 2: Oligonucleotides used in this work

Primer name	Template sequence	Sequence (5′-3′)
Tmar.gyrA.1	*T. maritima MBS8* genome	gtgagcaaaatgcgcgttgcggcagtcgacagcgatatatttatatagggatatagtaatagat aatatcacaggtggtatgaATGCCAGAGATCCTGATAAAC
Tmar.gyrA.2	*T. maritima MBS8* genome	tccattcataccacctgGGTATGTTCTATGGGTTTCC
Tmar.gyrB.1	*T. maritima MBS8* genome	catagaacatacccaggtggtatgaATGGAAAAGTACTCCGCTG
Tmar.gyrB.2	*T. maritima MBS8* genome	acgttcatcaaagttcatctagagcggccgCTAGATATCCAGTTCTTTCAC TTTC
pTNAg.GA.gyrAB.1	pLC70-gyrAB	tacgccaagcttggtaccgagctcgTCGACAGCGATATATTTATATAGG
pTNAg.GA.gyrAB.2	pLC70-gyrAB	tccattcataccacctGGGTATGTTCTATGGGTTTCC
pTNAg.GA.Y119F.1	pTNAg-gyrAB	tacgccaagcttggtaccgagctcgGTCGACAGCGATATATTTATATAG GG
pTNAg.GA.Y119F.2	pTNAg-gyrAB	gcctcgtgagtctCGCTTCCGTGAACCTCATC
pTNAg.GA.Y119F.3	pTNAg-gyrAB	gttcacggaagcgAGACTCACGAGGCTCGCAG
pTNAg.GA.Y119F.4	pTNAg-gyrAB	agcacactggcggccgttactagtgGGCTAGATATCCAGTTCTTTCACT TTC
RT-PCR.gyrA.1	pTNAg-gyrAB / pTNAg-Y119F	GTCGCGAAGAACACCTCATC
RT-PCR.gyrA.2	pTNAg-gyrAB / pTNAg-Y119F	TTGCCGAATCCCTTCTCTGT
RT-PCR.gyrB.1	pTNAg-gyrAB / pTNAg-Y119F	GCAAAACAGGCCAGAGACAG
RT-PCR.gyrB.2	pTNAg-gyrAB / pTNAg-Y119F	CACTTTCAGAGCGTGCCTTT
RT-PCR.gyrAB.1	pTNAg-gyrAB / pTNAg-Y119F	AGGGATTCGGCAAGAGAACA
RT-PCR.gyrAB.2	pTNAg-gyrAB / pTNAg-Y119F	TCCTCGACTTCCACACTTCC

Uppercase indicates identity to the template sequence ; lowercase indicates primer extension for Gibson assembly.

Primers Tmar.gyrA.1, Tmar.gyrA.2, Tmar.gyrB.1, Tmar.gyrB.2 were used to construct pLC70-gyrAB plasmid (unpublished data). pLC70-gyrAB was used as a template to construct pTNAg-gyrAB and pTNAg-Y119F.

Supplementary Table 3: Superhelical densities from various plasmids and viruses

Organism	Optimal growth temperature	Plasmid	Superhelical density	References
Escherichia coli	37	pTZ18	-0.051	Charbonnier F. and Forterre P., *J Bacteriol.* 1994
Escherichia coli	37	pBR322	-0.050	
Escherichia coli	37	M13mp19	-0.049	
Thermus sp. YS45	65	pTYS45-1	-0.057	
Rhodothermus marinus R21	65	pRM21	-0.063	
Thermus thermophilus HB8	80	pTT8	-0.059	
Halobacterium halobium GRB	37	pGRB	-0.068	
Haloferax volcanii WR11	37	pHV11	-0.068	
Haloferax volcanii WR12	37	pHV12	-0.060	
Halobacterium volcanii DS2	37	pHV2	-0.060	
Methanococcus sp. C5	30	pURB500	-0.058	
Methanosarcina acetivorans C2A	35	pC2A	-0.048	
Methanobacterium thermoautotrophicum Marburg DSM 2133	65	pME2001	-0.013	
Sulfolobus shibatae DSM 5389	78	pSSV1	+0.015	
Desulfurolobus ambivalens DSM 3772	80	pSL10	+0.007	
Pyrococcus abyssi GE5	95	pGT5	-0.003	
Thermococcus sp. GE31	80	pGN31	+0.035	López-García P. and Forterre P., *Mol. Microbiol.* 1997
Sulfolobus islandicus REN1H1	80	pRN1	+0.008	
Sulfolobus islandicus REN1H1	80	pRN2	+0.008	
Sulfolobus sp. NZ 54/3	80	pTAU4	+0.017	
Archaeoglobus profundus	80	pGS5	-0.033	Lopez-Garcia P. et al., *J Bacteriol*. 2000
SV40 virion	37 (host)	/	-0.051	Shure M. et al., *Nucleic Acids Res.* 1977
intracellular SV40		/	-0.050	
polyoma virion		/	-0.053	
intracellular polyoma		/	-0.050	
PM2 (marine bacteriophage)	37 (host)	/	-0.017	

Chlamydia trachomatis	37	pCT-L2	-0.07	Niehus E. et al., *J Bacteriol.* 2008
mammalian COS cells	37	pRSSVO	-0.0581	Tong W. et al., *J Mol Biol.* 2006
mammalian COS cells	37	pTEKO	-0.0583	
mammalian COS cells	37	pOS47	-0.0589	
mammalian COS cells	37	pOS67	-0.0556	
Saccharomyces cerevisiae	30?	pRSSVO	-0.0421	
Saccharomyces cerevisiae	30	TAC	-0.0511	Shen CH. et al., *Mol Cell Biol.* 2001
Saccharomyces cerevisiae	30	TA-HIS3	-0.0453	Kim Y, Clark DJ., *Proc Natl Acad Sci U S A.* 2002
Saccharomyces cerevisiae	30	TRP1ARS1	-0.0506	Pederson DS et al., *Proc Natl Acad Sci U S A.* 1986
Bacillus subtilis	37	pUB110	-0.074	calculated from: Nicholson WL, Setlow P., *J Bacteriol.* 1990
Mycobacterium tuberculosis	37	pSUM36	-0.060	García MT et al., *Front Microbiol.* 2018
Thermococcus nautili	87.5	pTN1	-0.002	Gorlas A. et al., *Extremophiles.* 2019
Sulfolobus sp. NZ 59/2	80	pSTHA	+0.006	Charbonnier F. and Forterre P., *J Bacteriol.* 1994
Thermotoga sp. RQ7	80	pRQ7	-0.067	Guipaud O. et al., *Proc Natl Acad Sci U S A.* 1997
Streptococcus pneumoniae TBB1	37	pLS1	-0.059	Ferrandiz et al., *Nucleic Acids Research.* 2016
Streptococcus pneumoniae TBB1 (SCN treated)	37	pLS1	-0.083	

Supplementary Table 4: Differential expression profile of pTNAg encoded genes

		**TKgyrAB vs TKY119**				**TKY119F vs TKAg**				**TKgyrAB vs TKAg**			
Locus name	Annotation	log2FC	Padj	NC (G)	NC(Y)	log2FC	Padj	NC (Y)	NC(A)	log2FC	Padj	NC (G)	NC(A)
gyrA	DNA gyrase subunit A	-0.11	0.52	40895	44113	NA	NA	NA	NA	NA	NA	NA	NA
gyrB	DNA gyrase subunit B	-0.07	0.80	30889	32426	NA	NA	NA	NA	NA	NA	NA	NA
*TK0149**	Pyruvoyl-dependent arginine decarboxylase	-0.18	0.37	15065	17049	**-0.43**	**0.0052**	17049	23037	**-0.61**	**4.88E-6**	15065	23037
Rep74	Rolling circle replication initiator protein	**-0.36**	0.13	2149	2560	-0.05	0.9	2765	2954	**-0.41**	**0.046**	2149	2954
p24	Orphan DNA binding protein	0.16	0.48	196	220	**-0.67**	**5.77E-6**	195	312	**-0.51**	**0.00033**	196	312
Kan	Kanamycin resistance gene	0.28	0.49	79	65	**-1.01**	**0.0003**	65	132	**-0.73**	**0.0056**	79	132
Amp	Ampicillin resistance gene	0.18	0.44	232	204	-0.25	0.26	204	243	-0.06	0.79	232	243

NC (G) – normalised counts TKgyrAB strain; NC (Y) normalised counts TKY119F strain; NC (A) - normalised counts TKAg; mean value of four replicates is given. NA – non applicable

The values above the significance threshold are indicated in bold letters (Padj ≤ 0.05 and Log2FC ≥ |0.33|).

* confers agmatine prototrophy to *T. kodakarensis*

Supplementary table 5: Expression profile of topologically relevant genes

			**TKgyrAB vs TKY119**				**TKY119F vs TKAg**				**TKgyrAB vs TKAg**			
Locus name	Old locus name	Annotation	log2FC	Padj	NC (G)	NC(Y)	log2FC	Padj	NC (Y)	NC(A)	log2FC	Padj	NC (G)	NC(A)
TK_RS02320	*TK0470*	reverse gyrase	0.25	**0.007**	27209	22822	0.23	**0.023**	22822	19434	**0.49**	**1.6E-09**	27209	19434
TK_RS02325	*TK0471*	TrmBL2	-0.12	0.580	19845	21549	0.30	0.070	21549	17478	0.18	2.3E-01	19845	17478
TK_RS02760	*TK0560*	Alba	0.30	0.122	41197	33370	0.31	0.126	33370	26977	**0.61**	**7.9E-05**	41197	26977
TK_RS03845	*TK0778*	Mini-A	0.05	0.749	5065	4892	0.16	0.146	4892	4368	0.21	**1.9E-02**	5065	4368
TK_RS03950	*TK0798*	DNA topoisomerase VI subunit A	-0.004	0.980	7165	7185	0.15	**0.045**	7185	6459	0.15	**2.2E-02**	7165	6459
TK_RS03955	*TK0799*	DNA topoisomerase VI subunit B	0.02	0.887	8763	8617	0.09	0.467	8617	8086	0.12	2.3E-01	8763	8086
TK_RS05360	*TK1091*	DNA topoisomerase III	-0.04	0.772	6031	6212	0.19	0.061	6212	5445	0.15	1.0E-01	6031	5445
TK_RS07015	*TK1413*	histone A	-0.29	0.296	12472	15259	**0.41**	0.107	15259	11501	0.12	6.6E-01	12472	11501
TK_RS11530	*TK2289*	histone B	0.19	0.401	9589	8377	0.30	0.151	8377	6818	**0.49**	**2.2E-03**	9589	6818
TK_RS05005	*TK1017*	chromosome segregation protein SMC	-0.07	0.815	15166	15965	0.08	0.782	15965	15078	0.008	9.8E-01	15166	15078

NC (G) – normalised counts TKgyrAB strain; NC (Y) normalised counts TKY119F strain; NC (A) - normalised counts TKAg; mean value of four replicates is given.

The values above the significance threshold are indicated in bold letters (Padj ≤ 0.05 and Log2FC ≥ 1.25).

Supplementary table 6: Fragments per kilobase million counts for annotated topoisomerases in *T. kodakarensis*

			**TKgyrAB (FPKM)**				**TKY119F**				**TKAg**			
Locus name	Old locus name	Annotation	Rep1	Rep2	Rep3	Rep4	Rep1	Rep2	Rep3	Rep4	Rep1	Rep2	Rep3	Rep4
N.A.	*N.A.*	GyrA	**1.41**	**1.44**	**1.58**	**1.56**	1.43	1.51	1.68	1.79	*N.A.*	*N.A.*	*N.A.*	*N.A.*
N.A.	*N.A.*	GyrB	**0.57**	**0.59**	**0.65**	**0.72**	0.57	0.60	0.66	0.80	*N.A.*	*N.A.*	*N.A.*	*N.A.*
TK_RS02320	*TK0470*	reverse gyrase	**0.47**	**0.45**	**0.43**	**0.53**	0.41	0.37	0.39	0.39	0.38	0.32	0.33	0.36
TK_RS03845	*TK0778*	Mini-A	0.51	0.46	0.47	0.52	0.53	0.46	0.46	0.43	0.45	0.47	0.42	0.42
TK_RS03950	*TK0798*	DNA topoisomerase VI subunit A	0.55	0.53	0.55	0.55	0.55	0.54	0.54	0.55	0.54	0.53	0.51	0.48
TK_RS03955	*TK0799*	DNA topoisomerase VI subunit B	0.50	0.46	0.44	0.43	0.48	0.45	0.44	0.40	0.48	0.44	0.42	0.41
TK_RS05360	*TK1091*	DNA topoisomerase III	0.14	0.14	0.13	0.16	0.14	0.14	0.15	0.15	0.14	0.13	0.12	0.15

N.A. not applicable

REFERENCES

1. Taboada,B., Estrada,K., Ciria,R. and Merino,E. (2018) Operon-mapper: a web server for precise operon identification in bacterial and archaeal genomes. *Bioinformatics*, **34**, 4118–4120.
